# Metabolic profiling of a transgenic *Caenorhabditis elegans* Alzheimer model

**DOI:** 10.1007/s11306-014-0711-5

**Published:** 2014-07-30

**Authors:** Roel Van Assche, Liesbet Temmerman, Daniel A. Dias, Berin Boughton, Kurt Boonen, Bart P. Braeckman, Liliane Schoofs, Ute Roessner

**Affiliations:** 1 Functional Genomics and Proteomics, Department of Biology, KU Leuven, Leuven, Belgium; 2 Metabolomics Australia, School of Botany, University of Melbourne, Melbourne, Australia; 3 Laboratory for Aging Physiology and Molecular Evolution, Department of Biology, Ghent University, Ghent, Belgium

**Keywords:** Metabolomics, Metabolic profiling, *Caenorhabditis elegans*, Alzheimer’s disease, Amyloid-*β*

## Abstract

Despite decades of research, no early-onset biomarkers are currently available for Alzheimer’s disease, a cureless neurodegenerative disease afflicting millions worldwide. In this study, transgenic *Caenorhabditis elegans* were used to investigate changes in the metabolome after induced expression of amyloid-β. GC- and LC–MS-based platforms determined a total of 157 differential features. Some of these were identified using *in*-*house* (GC–MS) or public libraries (LC–MS), revealing changes in allantoin, cystathionine and tyrosine levels. Since *C. elegans* is far better suited to metabolomics studies than most other model systems, the accordance of these findings with vertebrate literature is promising and argues for further use of *C. elegans* as a model of human pathology in the study of AD.

**Electronic supplementary material:**

The online version of this article (doi:10.1007/s11306-014-0711-5) contains supplementary material, which is available to authorized users.

## Introduction

Alzheimer’s disease (AD) is the most common cause of dementia, accounting for over 70 % of all cases worldwide (World Alzheimer’s Report, 2009). In addition, AD causes a severe social and financial impact on patients and their environment. The number of patients worldwide with AD is estimated at a current 24 million, a number that will have tripled by 2050 (World Health Organization, 2012). AD is a progressive disease with a long preclinical phase of 10–15 years (Tarawneh and Holtzman 2012), which creates opportunities for a biomarker detection approach. AD is characterized by inflammation, neuronal loss, intracellular aggregation of the protein tau and extracellular plaques of the peptide amyloid-*β*. Amyloid-*β*, generated from the amyloid-*β* precursor protein (APP), is mainly processed in an anti-amyloidogenic manner (Haass et al. 2012). However, in AD patients, APP is overly processed according to the amyloidogenic pathway, which leads to the release of aggregating amyloid peptides (Haass et al. 2012). Additionally, the microtubule stabilizing protein tau becomes hyperphosphorylated and forms intracellular neurofibrillary tangles (Mandelkow and Mandelkow 2012). Since there is no cure for AD, it is becoming increasingly more important to find early pathological markers which could easily be measured by a non-invasive method, prior to the emergence of clinical symptoms.

Current biomarkers in cerebrospinal fluid (CSF) are the amount of total tau, phosphorylated tau, amyloid-β (Blennow et al. 2012) and the 42/40 ratio of amyloid-β (Wiltfang et al. 2007). Although CSF removal is an uncomfortable, invasive procedure, it is often used to diagnose AD. A range of imaging techniques has been developed over the last couple of years to improve the diagnosis of AD (Johnson et al. 2012). Despite the improvement of fluid biomarker discovery and imaging techniques, AD is typically diagnosed when patients start displaying cognitive impairment. Recently, researchers have started turning towards metabolomics as a promising method to assist in the search for early biomarkers of AD (Trushina and Mielke 2013).

Metabolomics uses a range of sensitive and complementary analytical platforms to study the levels of small molecules in cells, tissues, bio-fluids and entire organisms, referred to as the metabolome (Roessner and Bowne 2009). Because gene expression, protein activity and environment all exert certain influences on the metabolome, metabolomic readouts closely reflect cellular processes and provide highly accurate snapshots of an organism’s state. Two platforms are mainly used: gas or liquid chromatographic separation hyphenated to mass spectrometry (GC–MS and LC–MS) and nuclear magnetic resonance (NMR)-based spectroscopy (Temmerman et al. 2013). Current literature on metabolomics related to AD is rather limited and a distinct set of metabolic markers has not yet been discovered (Graham et al. 2013a, b; Kaddurah-Daouk et al. 2011, 2013; Lin et al. 2013; Mapstone et al. 2014; Motsinger-Reif et al. 2013; Orešič et al. 2011; Salek et al. 2010; Sato et al. 2012; Trushina et al. 2013). This may in part be due to the underlying heterogeneity of the sample groups. To improve this issue, the use of a model can be advantageous.

Model organisms are used to investigate the function of certain factors in a simplified system in comparison to humans. Many model organisms have been used in the study of AD (e.g. *Saccharomyces cerevisiae* (De Vos et al. 2011), *Caenorhabditis elegans* (Link 2006), *Drosophila melanogaster* (Iijima-Ando and Iijima 2010), *Mus musculus* (Elder et al. 2010). Here, *C. elegans* was chosen because it allows for the most stringent level of experimental control in the study of multicellular organisms. This addresses the importance of minimizing unwanted variation, especially necessary when using sensitive techniques like metabolomics, biomarker discovery and compound screens. A transgenic, temperature-sensitive strain expressing amyloid-*β* in the neurons was selected, enabling time- and site- controlled expression of the transgene. Such strains have already proven their value in AD research in the study of amyloid-*β* aggregation (Fay et al. 1998), gene expression (Link 2003), toxicity screening (Dostal and Link 2010), learning behavior (Dosanjh et al. 2010) and proteomic changes (Boyd-Kimball et al. 2006). In this study, a metabolic fingerprint was generated of a well-established (Boyd-Kimball et al. 2006; Dosanjh et al. 2010; Dostal and Link 2010; Link 1995, 203) transgenic AD strain in order to monitor metabolic changes due to expression of amyloid-*β*. Both non-targeted GC–MS and LC–MS analyses were performed, ensuring a broad detection of the extracted metabolites. LC–MS analysis was further refined using two different chromatographic separation methods (reversed phase (RP) and aqueous normal phase (ANP)). Because *C. elegans* can be used to screen a large amount of metabolites in a relatively short time, these findings will form the basis of future testing of drug efficiency and the mode-of-action during AD progression.

## Materials and methods

### *C. elegans* culture and sampling

**Fig. 1:**
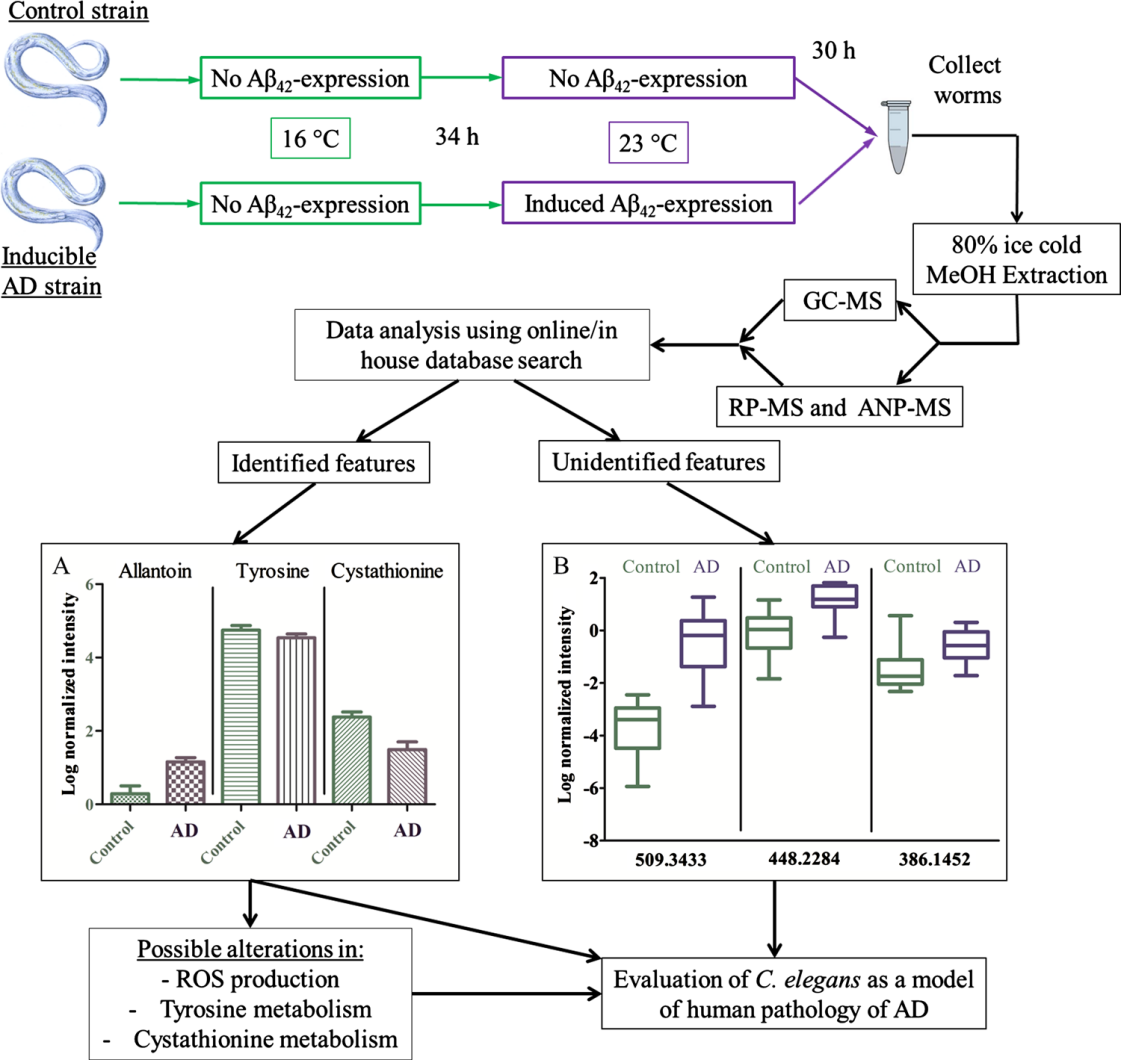
Scheme of experimental setup. Both AD model and control strains were cultured for 34 h at 16 °C. After this period, a temperature upshift to 23 °C was performed, inducing amyloid-β_42_ expression in the AD strain only. Thirty hours later, worms were collected and snap frozen with liquid nitrogen. An 80 % ice-cold methanol extraction was performed and samples were aliquoted for GC–MS, RP-QTOF or ANP-QTOF. **A** After data-analysis, features that were putatively identified showed a similar pattern as seen in previous human AD research. **B** Some of our unidentified features could be linked to LC–MS AD metabolomics profiling literature

Temperature-sensitive transgenic (CL2355) and control (PD8120) strains were kindly provided by Professor Christopher Link (University of Colorado at Boulder, USA). Strains were cultured at 16 °C on standard nematode growth medium (NGM) agar seeded with *Escherichia coli* OP50 bacteria (Brenner, 1974). CL2355 (*smg*-*1*(cc546);dvIs50 [pCL45 (*Psnb*-*1*::Aβ_1-42_::3′ UTR(long) + *Pmtl*-*2*::GFP]) drives pan-neuronal expression of the peptide Aβ_1–42_ under control of the *C. elegans* synaptobrevin (*snb*-*1*) promoter. The expression can be induced by a temperature upshift from 16 to 23 °C (Fig. 1), as a result of the temperature-sensitive *smg*-*1* ^*ts*^ background, also present in the control strain, in which nonsense-mediated mRNA decay (NMD) is disturbed upon temperature upshift (Link 2003).

Age-synchronized transgenic and control strains were cultured in liquid medium with *E. coli* K12 as a food source, for 34 h at 16 °C. Food availability was held constant (OD_600_ = 1.68) by adding bacteria twice a day during culturing. After 34 h, both strains were shifted to 23 °C, inducing the expression of Aβ_1-42_ in the AD strain only. Another 30 h later, worms were collected by pelleting on ice (Fig. 1). The removal of bacteria and debris was carried out using a sucrose-flotation (60 % sucrose). After collection, the worms were washed with S-buffer (Brenner 1974) for 5 times and partitioned into aliquots of maximum 0.3 ml per tube (Precellys lysing kit, Bertin Technologies). The aliquots were snap frozen in liquid nitrogen and stored at −80 °C.

A total of 14 biologically independent samples for each condition were used. As negative controls, three samples of each condition without temperature upshift were produced, in addition to four supernatant samples. The latter were used to establish the metabolite baseline of the medium: features extracted from these negative controls were omitted from all further sample analyses.

### Extraction

We opted for an extraction using ice-cold 80 % methanol, as described earlier for *C. elegans* (Geier et al, 2011). Ice-cold methanol, containing an external standard (^13^C_6_-sorbitol (0.5 mg ml^−1^), ^13^C_5_^15^N-Valine (0.5 mg ml^−1^), 2-aminoanthracene (0.25 mg ml^−1^) and pentafluorobenzoic acid (0.25 mg ml^−1^), was added to the worm pellet until 80 % methanol was reached. All samples were homogenized at low temperature (Precellys 24, Bertin Technologies; 2 × 30 s, 4800 rpm, −20 °C) to avoid potential metabolite degradation. Subsequently, samples were held on ice for 15 min and were then centrifuged for 15 min at 13,000 rpm. Supernatants were transferred to new Eppendorf tubes and pellets were re-extracted with 80 % methanol. To avoid protein contamination, all samples were filtered using a 3 kDa cutoff filter (Amicon Ultra UFC500308). Both supernatants were combined and re-divided into 3 aliquots for GC–MS and LC–MS (RP and ANP coupled to quadrupole time of flight mass spectrometry (Q-TOF)) analyses. These aliquots were dried using a speed-vacuum concentrator and then stored at −80 °C until analysis.

### GC–MS analysis

The dried samples were redissolved in 10 μl of 30 mg ml^−1^ methoxyamine hydrochloride in pyridine and derivatised at 37 °C for 120 min with mixing at 500 rpm. The samples were then treated for 30 min with 20 μl *N*,*O*-*bis*-(trimethylsilyl)trifluoroacetamide (BSTFA) and 2.0 μl retention time standard mixture [0.029 % (*v*/*v*) *n*-dodecane, *n*-pentadecane, *n*-nonadecane, *n*-docosane, *n*-octacosane, *n*-dotriacontane, *n*-hexatriacontane dissolved in pyridine] with mixing at 500 rpm. Each derivatised sample was allowed to rest for 60 min prior to injection.

Samples (1 μl) were injected into a GC–MS system comprised of a Gerstel 2.5.2 autosampler, a 7890A Agilent gas chromatograph and a 7000 Agilent triple-quadrupole MS (Agilent). The MS was adjusted according to the manufacturer’s recommendations using *tris*-(perfluorobutyl)-amine (CF43). The GC was performed on a 30 m VF-5MS column with 0.2 μm film thickness and a 10 m Integra guard column (J & W, Agilent). The injection temperature was set at 250 °C, the MS transfer line at 280 °C, the ion source adjusted to 250 °C and the quadrupole at 150 °C. Helium was used as the carrier gas at a flow rate of 1.0 ml min^−1^. For the polar metabolite analysis, the following temperature program was used; start at injection 70 °C, a hold for 1 min, followed by a 7 °C min^−1^ oven temperature ramp to 325 °C and a final 6 min heating at 325 °C in which the data was acquired in full-scan mode. Both chromatograms and mass spectra were evaluated using AMDIS (NIST, www.chemdata.nist.gov) and Agilent MassHunter Workstation Software, Quantitative Analysis, Version B.05.00/Build 5.0.291.0 for GCMS. Mass spectra of eluting compounds were identified using NIST08 database and the *in*-*house* Metabolomics Australia mass spectral library. All matching mass spectra were additionally verified by determination of the retention time and index in comparison to those of standard substances. Every six samples, a pooled instrument control sample consisting of 54 standard compounds was run to evaluate potential retention time shifts and loss in sensitivity (Supplemental Fig. 1). As can be expected for GC, all quality control runs overlapped clearly and no significant retention time shift was present. A significant loss in sensitivity was not observed. It can therefore be taken into account by normalization strategies (see below). None of the differential and identified metabolites had multiple TMS derivatives. All data were exported as a comma separated value file for further data analysis.

### LC–MS analysis

#### Materials

LC–MS grade formic acid and ammonium acetate were purchased from Sigma-Aldrich (Sydney, Australia). Deionized water (18.2 MΩ) was used throughout all experiments. HPLC grade methanol and acetonitrile (ACN) were purchased from Burdick and Jackson (Ajax, Sydney, Australia). Reversed phase (RP) chromatography was done using a Zorbax Eclipse XDB-C18 m 2.1 × 100 mm, 1.8 μm (Agilent, Santa Clara, CA, USA). The Cogent diamond hybrid 2.1 × 100 mm, 4 μm particle size ANP column was purchased from MicroSolv Technology (Brisbane, Australia).

#### LC–MS system

In this experiment an Agilent 1200 series HPLC was used (Santa Clara, CA, USA) comprising of a vacuum degasser, binary pump, thermostated auto sampler and column compartment. Extraction procedures, solvent gradients, concentration sample, column conditions and mass spectrometer settings were optimized using pooled samples; reflecting an averaged sample of the overall experiment. The settings found to be optimal for *C. elegans* metabolites were then used for all subsequent runs. For RP chromatography, a 10 min linear gradient of 95:5 water/ACN to 5:95 water/ACN at 0.4 ml min^−1^ was used while the column temperature was held at 50 °C. Both mobile phases contained 0.1 % formic acid. For the complementary ANP procedure, solvents were made with uttermost care and the system was thoroughly flushed to ensure a proper separation of the metabolites. The organic mobile phase solvent (B) was composed of 90 % ACN with 0.1 % (w/v) ammonium acetate and 0.1 % acetic acid. The aqueous mobile phase (A) was composed of 100 % deionized water with 0.1 % (w/v) ammonium acetate and 0.1 % acetic acid (pH 3.4). The column flow-rate was 0.4 ml min^−1^ and column temperature was kept at 50 °C. The optimal gradient started at 100 % B then linearly decreased to 40 % B over 10 min, followed by a 1 min hold at 40 % B. The column was then re-equilibrated at 100 % B for 6 min^20^. For both modes, a washing step was added every run to control for unwanted carry-over. Every six samples, a pooled biological control sample was run to evaluate potential retention time shifts and variations in mass accuracy (Supplemental Fig. 2). Retention time shifts were never bigger than 0.1 min and the average deviation of the mass accuracy always remained lower than 1.78 ppm (Supplemental Table 1).

The mass spectrometer used was an Agilent 6520 QTOF MS system (Santa Clara, CA, USA) with a dual spray ESI source. The conditions for the source were: nebulizer pressure of 45 psi, gas flow-rate of 10 l min^−1^, gas temperature 300 °C, capillary voltage of 4 kV and skimmer 65 V. Measurements were performed in the extended dynamic range mode (m/z range of 70–1700 amu), both in positive and negative ion mode and collecting centroid data. Data were exported as.mzdata to be further analyzed in MZmine 2.10 (Pluskal et al. 2010). In addition, to increase the accuracy of the identification, high resolution (70,000), more accurate (<3 ppm) MS and MS/MS data of differential features were obtained by running pooled samples using a ‘top 10’ method on a Q Exactive Hybrid Quadrupole-Orbitrap mass spectrometer (Thermo Scientific).

LC–MS data analysis was performed using MZmine 2.10 (Pluskal et al. 2010). After centroid peak detection, all data points above the noise level were processed as pairs of *m/z* and intensity values. Peak lists were created using the chromatogram builder. The chromatograms were deconvoluted and isotopic peaks were grouped. Finally, peak lists were aligned using the random sample consensus (RANSAC) alignment method (Pluskal et al. 2010). After filtering and gap filling, the data matrix was exported as a comma separated value file for further processing. All parameters were optimized for each data collection mode. LC–MS identification was performed using public databases (HMDB, KEGG and Metlin). Based on mass value (Δppm < 10 ppm) and accurate mass (Δppm < 3 ppm) features were matched against these databases.

### Data analysis

Statistical analysis was performed using the *R* package *metabolomics* (De Livera et al. 2013) and the MetaboAnalyst webserver (Xia et al. 2012). An initial log_2_ transformation was applied to obtain a normal distribution. After this transformation, the dataset was median normalized and a combination of multivariate and univariate statistical tests was performed. Principal components analysis (PCA), an unsupervised explorative data analysis method, was performed to evaluate the overall variance in the obtained datasets. Similarly, supervised partial-least squares discriminant analysis (PLS-DA) was conducted to better explore the variance differentiating the two experimental conditions (AD vs. control). Variable Importance in Projection (VIP) scores represent which of the features contribute most to the differentiation of the experimental groups in PLS-DA analysis. Significant differences in abundance of individual features between conditions were evaluated using a standard *t* test. All *p* values were adjusted according to the Benjamini & Hochberg principle to take false discovery rate into account.

## Results and discussion

There are already a few reports on the metabolome of human AD cerebrospinal fluid (CSF) (Kaddurah-Daouk et al. 2011, 2013; Orešič et al. 2011; Trushina et al. 2013) (Graham et al. 2013a), plasma (Trushina et al. 2013) and of CSF reflecting the pathological progress from mild cognitive impairment to AD (Orešič et al. 2011). Despite these research efforts, working with human samples implies a high level of inherent variation (age, sex, diet, medical history, etc.), which may mask relevant results. Therefore, use of more controllable model organisms can help to deliver a more delineated fingerprint, which can then be used for targeted studies in patients. Based on this reasoning, metabolic analyses on transgenic AD mice (Fukuhara et al. 2013; Graham et al. 2013b; Salek et al. 2010) revealed a widespread perturbation of metabolism in different tissues and bio fluids. In the same vein, here *C. elegans* was used to discover metabolic changes due to AD by generating a metabolic fingerprint of a transgenic, pan-neuronal amyloid-*β* strain.

*Caenorhabditis elegans* is a suitable model organism to screen multiple drug compounds efficiently and has the potential to discover markers for diseases in a cheap, fast and controlled manner. Metabolomics has proven its value for *C. elegans* research (Fuchs et al. 2010; Hughes et al. 2009), although profiling, to our knowledge has not been performed with transgenic *C. elegans* AD models. We used a metabolic approach to evaluate the *C. elegans* amyloid-*β* AD model, relying on a combination of GC–MS and four LC–MS platforms (ANP-MS and RP-MS, each acquired in positive and negative ion mode).

### Overall feature detection and sample separation

**Fig. 2:**
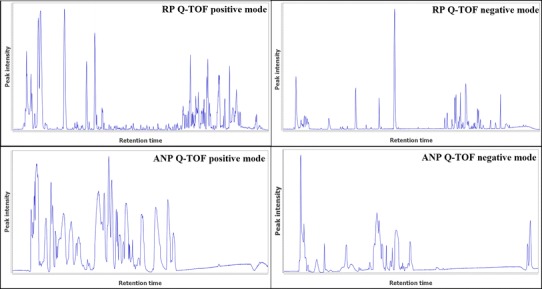
Representative UPLC ESI-base peak chromatograms of *C. elegans* extracts from different platforms. *X*-axis: Retention time (from 0 to 14 min), *Y* axis: Peak intensity (total ion count from 0 to 8.0E6)

A clear chromatographic separation was achieved for all approaches (Fig. 2), resulting in a final total of 157 differential features (*p* value <0.05) (Table 1). Mean normalized abundance and standard deviation of all samples in all modes were determined (Supplemental Table 2).

**Table 1:** Total of differential features in different modes

Differential features different modes		
Method	Ion mode	Differential features
LC–MS: RP	+	14
LC–MS: RP	−	113
LC–MS: ANP	+	21
LC–MS: ANP	−	1
GC–MS	+	8

*LC* liquid chromatography, *RP* reversed phase, *ANP* aqueous normal phase, *GC* gas chromatography)

Unsupervised multivariate statistics did not always succeed in separating the experimental groups (Supplemental Figs. 3, 4). Principal components analysis (PCA) generally resulted in a modest separation of the experimental groups. Poor PCA plot separations are also the case in similar studies (Lin et al. 2013; Trushina et al. 2013), but these rely on less controllable model systems. This could possibly be explained by the sample type: as opposed to human CSF or brain tissue samples, we used whole-mount extracts for analysis. Therefore, the behavior of some differentiating features might yet be diluted or masked. Even though the central nervous system is the actual site of interest, it can currently not be distinguished from other tissues in *C. elegans*. Nevertheless, partial least squares discriminant analysis (PLS-DA) in combination with variable importance in projection tables (Supplemental Figs. 5–11) illustrate that observed differential features considerably contribute to the separation of the two groups. Evaluation of volcano plots, showing the significance and fold-changes of all data points, was also indicative of a defined set of robustly differential features (Supplemental Figs. 12–16).

### Differential features as a result of AD pathology in *C. elegans*

For the GC approach every peak corresponding to a feature was manually selected and compared to a library of reference compounds, resulting in a final list of 76 unique compounds. Upon differential analysis, only a few known metabolites were significantly altered in AD conditions as compared to controls (Table 2). Of all features, 38 % could not be identified because of a lack of corresponding library entry (in-house or NIST08) (Supplemental Fig. 17).

**Table 2:** Significant GC–MS (*p* value <0.05) features with mass identifiers, retention time, *p* value (*adjusted according to the Benjamini & Hochberg principle), Z-factor, fold change and identity

GC–MS differential features					
Mass identifiers	RT	*p* value*	Z-factor	Fold change	Identity
188	15.1	0.0253	−1.75	3.33	Unknown
314, 329	15.5	0.0253	−3.28	1.93	Unknown
411	31	0.0253	−6.2	0.56	Unknown
299	16.2	0.0253	−3.51	1.88	Unknown
403, 189	17.2	0.0253	−3.04	0.53	Unknown
264, 279	20.8	0.0253	−3.06	1.83	Allantoin
278, 245	25.3	0.0253	−3.34	0.54	Cystathionine
243, 128	15	0.0481	−3.83	1.58	Unknown
218, 280	21.8	0.0022	−11.09	1.35	Tyrosine

Z-factor provides a useful tool for comparison and evaluation of the quality of the assay (Zhang 1999)

LC–MS data were acquired in positive and negative ion mode, each combined with two separation methods (RP and ANP) (Fig. 2). As such, an elaborate spectrum of metabolites could be examined. RP and ANP methodologies are suitable for differing sub-populations of the metabolome, explaining why only a few features were seen in both approaches. Features detected in both ANP and RP delivered similar readouts (Supplemental Fig. 18), providing corroborative evidence for the observed results. LC–MS profiling resulted in a detection of 2,096–11,039 features, depending on the acquisition mode (ANP-MS negative mode: 2096; ANP-MS positive mode: 4424; RP-MS negative mode: 5992; RP-MS positive mode: 11039). It is immediately clear that the RP negative mode dataset shows the highest amount of differential features (Table 1, Supplemental Table 3). By looking in further detail to this mode, many features were observed to co-elute and have similar fold changes. This may imply that a single metabolite may be present as a number of adducts which could lead to deconvolution of several related features. Therefore, an adduct search using the CAMERA package was performed on RP negative mode data (Kuhl et al. 2012). Approximately 15 % of the features were identified as adducts. The most significant differential (*p* value <0.01) features are presented in Table 3, remaining differential results are shown in supplemental Table 3. In sum, LC–MS analysis revealed 149 differential features, mostly detected from reversed phase LC in combination with negative ion mode.

**Table 3:** Features related to AD pathology from LC–MS analysis (*p* value <0.01)

LC–MS differential features					
m/z value	RT	*p* value*	Z-factor	Fold change	Mode
180.065	1.3	0.0446	−2.63	1.36	RP−
Identified as tyrosine (C_9_H_11_NO_3_, [M–H]–)					
173.1108	3.1	0.0017	−1.38	2.11	ANP+
131.1159	7	0.0017	−1.07	4.76	ANP+
169.0724	7	0.0017	−1.1	3.21	ANP+
230.1783	8.1	0.0017	−0.19	7.92	ANP+
384.1027	1.4	0.0037	−2.06	0.57	RP−
509.3433^a^	12.4	0.0037	−1.1	9.54	RP−
929.6274′	11.6	0.005	−1.35	15.77	RP−
464.5713′	11.6	0.005	−1.83	5.48	RP−
366.1274	12.7	0.005	−1.71	9.96	RP−
929.8786	3.8	0.005	−0.72	4.03	RP−
114.0901	0.8	0.0052	−1.37	2.91	RP+
448.2284	9.7	0.0052	−1.89	2.35	RP+
131.1167	0.8	0.0061	−1.36	3.56	RP+
362.2059	8.8	0.0061	−2.09	2.56	RP+
131.2164	0.8	0.0061	−1.62	2.72	RP+
485.1334	4.4	0.0061	−1.3	7.21	RP+
516.0773	3.7	0.0061	−1.77	3.87	RP+
131.0899	0.8	0.0068	−1.84	2.85	RP+
397.2089	8.5	0.0068	−2.05	3.35	RP+
173.1239	3	0.0084	−0.69	3.39	ANP+
449.1221	8	0.0096	−2.1	6.72	RP−

m/z value, retention time, *p* value (*adjusted according to the Benjamini and Hochberg principle), Z-factor, fold change, separation-detection mode and putative similarities with literature are provided. For tyrosine, formula and adduct ion are also shown. Z-factor provides a useful tool for comparison and evaluation of the quality of the assay (Zhang 1999). Adducts were combined and indicated with ′

^a^Confirmed in Lin et al. 2013. Accurate mass of tyrosine in Supplemental Table 4

By comparing the obtained results with research on AD LC–MS metabolomics literature (Lin et al. 2013; Trushina et al. 2013), some putative features were found with matching mass (Δppm < 50 ppm) and similar fold changes (Fig. 1; Table 3, Supplemental Table 3). These confirm that the same reactions might be (de)activated in *C. elegans* when comparing to mouse or human. Since most metabolomics experiments conducted to date relied on targeted methods, it can be assumed that this number is an underestimation of the actual correspondence (i.e. when comparing to other non-targeted studies). In addition, more corresponding features can be expected if not only amyloid-β_1–42_, but also the protein tau would be expressed in the *C. elegans* model. Amyloid-β_1–42_ probably only induces a part of the metabolic changes which occur in AD (Ittner and Götz 2011), therefore, adding tau might better reflect the biochemical changes related to AD progression.

### Known metabolic markers of AD

In our *C. elegans* experiments, several putatively identified metabolites correspond to metabolites previously associated with AD pathology (Tables 2, 3). These are of special interest for further discussion. A higher level of allantoin was observed in our experiments in *C. elegans*, matching observations in human plasma and mice (Fukuhara et al. 2013; Zitnanová et al. 2004). Allantoin is produced in a non-enzymatic oxidation reaction when uric acid is exposed to reactive oxygen species (ROS). This is further supported by evaluation of the GC–MS VIP scores (Supplemental Fig. 7), which are high for both uric acid and allantoin itself, indicating these features contribute strongly to the differentiation of both conditions. Allantoin is often used as an oxidative marker (Yardim-Akaydin et al. 2006). Oxidative stress is a frequently discussed topic in AD research, since it may precede the appearance of pathological hallmarks, e.g. senile plaques and neurofibrillary tangles (Perry et al. 2002). Oxidative stress in AD is probably the result of a disturbed redox balance due to malfunctioning of the mitochondria (Zhao and Zhao 2013). Both amyloid-β and tau can be found in the mitochondria where they dysregulate the oxidative phosphorylation system (complex IV and I, respectively) (Rhein et al. 2009), associated with an increased ROS production. This could then indeed set the scene for the observed increase in allantoin levels.

Upon expression of amyloid-β, tyrosine was upregulated in both LC- and GC–MS analyses, indicating the robustness of this result. Alterations of the tyrosine pathway in CSF, serum and autopsy-confirmed brain tissue of AD patients were also previously observed (Kaddurah-Daouk et al. 2011; Trushina et al. 2013). Tyrosine is an important precursor of the neurotransmitter dopamine, and of the catecholamines norepinephrine and epinephrine. When dopamine is formed, tyrosine is processed by tyrosine hydroxylase (TH). A reduced activity of TH (Trillo et al. 2013) and norepinephrine/epinephrine (Kaddurah-Daouk et al. 2011) have been observed in AD patients. The here observed upregulation of tyrosine might therefore be due to a reduced activity of TH, but this remains to be confirmed.

A decreased cystathionine concentration was observed after the expression of amyloid-β in *C. elegans*. Polymorphisms in cystathionine beta synthase (CBS), catalyzing the conversion of homocysteine to cystathionine, are well-known risk factors for AD (Perluigi and Butterfield 2012). These gene polymorphisms are known to decrease CBS activity and cause a high concentration of homocysteine and a low concentration of cystathionine (Bi et al. 2010), in line with our observations in *C. elegans*.

The clear correlations of these identified, differential metabolites with vertebrate AD pathology support the robustness of the *C. elegans* model system.

### Long-lived *C. elegans* exhibit opposite alterations

Because *C. elegans* is also a well-established model system for aging research, there is added value in comparing information for long-lived, healthy strains with the here used AD strain. This is because the latter is hallmarked by a decreased lifespan and impaired learning behavior due to neurodegeneration (Dosanjh et al. 2010), therefore displaying opposite phenotypes. Proteomic analysis (Depuydt et al. 2014) of the long-lived *daf*-*2* mutant revealed an increase in tyrosine catabolism. This contrasts with the higher levels of tyrosine observed in the AD model used here. Similarly, a strong upregulation of CBS is observed in the long-lived *daf*-*2* mutant (Depuydt et al. 2014), implying increased concentrations of cystathionine. This again confirms the molecular basis for the opposite phenotypes. As more and more-omics data are becoming available, such comparisons could in the future assist in discriminating general, aging-related effects from more AD-specific perturbations.

### Prospects

Although our results are promising and some comparisons with human AD pathology could be made, elaborate comparison of studies is not straightforward. This has a dual reason: for one, human studies rely on very distinct sample types. Brain tissue, on one hand, can only be used from post-mortem patients and often suffers from degradation. CSF from patients, on the other hand, is an achievable alternative, but implies a change towards indirect results from a biofluid, rather than direct information from the affected tissue. In addition, limited effort is made for the complete determination of the metabolic fingerprint. This is readily understood from the set of metabolic markers discussed above, which do not (yet) display any clear biological coherence or pathway logic. If we want to accurately map the pathological process, complete identified metabolic fingerprints—which represent the comprehensive status of all extracted metabolites—should be compared over time in profiling experiments. This strategy will allow for the robust discovery of potential biomarker candidates and grants an invaluable advantage to complementary compound screens. After administration of a certain lead compound, the fingerprint can indicate which (sub)processes are altered. Currently, essential information can easily be overlooked due to the partial identification of the fingerprints. Such analyses should ideally be performed preclinical, in a controlled model where metabolic fingerprints can be identified more easily.

### Concluding remarks

Our results show that *C. elegans* has the abilities to develop into an amenable model for AD metabolomics experiments. The here described set of metabolites provide a blueprint for future completion of the AD fingerprint, as such further refining our mechanistic insights into this devastating disease. Metabolomic analyses, compound screenings and biomarker discovery require an exceptional high level of experimental control. Future experiments with optimized double transgenic worms, expressing amyloid-*β* and tau together, will therefore be invaluable to assist in the advances of metabolomics with regards to AD progression.

## Electronic supplementary material

Below is the link to the electronic supplementary material.

Supplementary material 1 (DOCX 16892 kb)

## References

[CR1] Bi X-H, Zhao H-L, Zhang ZX (2010). Association analysis of CbetaS 844ins68 and MTHFD1 G1958A polymorphisms with Alzheimer’s disease in Chinese. Journal of Neural Transmission.

[CR2] Blennow K, Zetterberg H, Fagan AM (2012). Fluid biomarkers in Alzheimer’s disease. Cold Spring Harbor Perspectives in Medicine.

[CR3] Boyd-Kimball D, Poon HF, Lynn BC (2006). Proteomic identification of proteins specifically oxidized in *Caenorhabditis elegans* expressing human Abeta(1-42): Implications for Alzheimer’s disease. Neurobiology of Aging.

[CR4] Brenner S (1974). The genetics of *Caenorhabditis elegans*. Genetics.

[CR5] De Livera AM, Olshansky M, Speed TP (2013). Statistical analysis of metabolomics data. Methods in Molecular Biology.

[CR6] De Vos A, Anandhakumar J, Van den Brande J (2011). Yeast as a model system to study tau biology. International Journal of Alzheimer’s Disease.

[CR7] Depuydt G, Xie F, Petyuk VA (2014). LC-MS proteomics analysis of the insulin/IGF-1-deficient *Caenorhabditis elegans* daf-2(e1370) mutant reveals extensive restructuring of intermediary metabolism. Journal of Proteome Research.

[CR8] Dosanjh LE, Brown MK, Rao G (2010). Behavioral phenotyping of a trangenic *C. elegans* expression neuronal amyloid beta. Journal of Alzheimer’s Disease.

[CR9] Dostal V, Link CD (2010). Assaying β-amyloid toxicity using a transgenic *C. elegans* model. Journal of Visualized Experiments.

[CR10] Elder GA, Sosa MAG, De Gasperi R (2010). Transgenic mouse models of Alzheimer’ s disease. Mount Sinai School of Medicine.

[CR11] Fay DS, Fluet A, Johnson CJ (1998). In vivo aggregation of beta-amyloid peptide variants. Journal of Neurochemistry.

[CR12] Fuchs S, Bundy JG, Davies SK (2010). A metabolic signature of long life in *Caenorhabditis elegans*. BMC Biology.

[CR13] Fukuhara K, Ohno A, Ota Y (2013). NMR-based metabolomics of urine in a mouse model of Alzheimer’s disease: Identification of oxidative stress biomarkers. Journal of Clinical Biochemistry and Nutrition.

[CR14] Geier FM, Want EJ, Leroi AM (2011). Cross-platform comparison of *Caenorhabditis elegans* tissue. Analytical Chemistry and Bioanalytical Chemistry.

[CR15] Graham SF, Chevallier OP, Roberts D (2013). Investigation of the human brain metabolome to identify potential markers for early diagnosis and therapeutic targets of Alzheimer’s disease. Analytical Chemistry.

[CR16] Graham SF, Holscher C, McClean P (2013). 1H NMR metabolomics investigation of an Alzheimer’s disease (AD) mouse model pinpoints important biochemical disturbances in brain and plasma. Metabolomics.

[CR17] Haass C, Kaether C, Thinakaran G (2012). Trafficking and proteolytic processing of APP. Cold Spring Harbor Perspectives in Medicine.

[CR18] Hughes SL, Bundy JG, Want EJ (2009). The metabolomic responses of *Caenorhabditis elegans* to cadmium are largely independent of metallothionein status, but dominated by changes in cystathionine and phytochelatins research articles. Journal of Proteome Research.

[CR19] Iijima-Ando K, Iijima K (2010). Transgenic *Drosophila* models of Alzheimer’s disease and tauopathies. Brain Structure & Function.

[CR20] Ittner LM, Götz J (2011). Amyloid-β and tau: A toxic pas de deux in Alzheimer’s disease. Nature.

[CR21] Johnson KA, Johnson KA, Fox NC, Sperling RA (2012). Brain imaging in Alzheimer’s disease. Cold Spring Harbor Perspectives in Medicine.

[CR22] Kaddurah-Daouk R, Rozen S, Matson W (2011). Metabolomic changes in autopsy-confirmed Alzheimer’s disease. Alzheimer’s & Dementia.

[CR23] Kaddurah-Daouk R, Zhu H, Sharma S (2013). Alterations in metabolic pathways and networks in Alzheimer’s disease. Translational Psychiatry.

[CR24] Kuhl C, Tautenhahn R, Böttcher C (2012). CAMERA: An integrated strategy for compound spectra extraction and annotation of liquid chromatography/mass spectrometry data sets. Analytical Chemistry.

[CR25] Lin S, Liu H, Kanawati B (2013). Hippocampal metabolomics using ultrahigh-resolution mass spectrometry reveals neuroinflammation from Alzheimer’s disease in CRND8 mice. Analytical and Bioanalytical Chemistry.

[CR26] Link CD (1995). Expression of human beta-amyloid peptide in transgenic *Caenorhabditis elegans*. Proceedings of the National Academy of Sciences of the United States of America.

[CR27] Link C (2003). Gene expression analysis in a transgenic *Caenorhabditis elegans* Alzheimer’s disease model. Neurobiology of Aging.

[CR28] Link CD (2006). *C. elegans* models of age-associated neurodegenerative diseases: Lessons from transgenic worm models of Alzheimer’s disease. Experimental Gerontology.

[CR29] Mandelkow E-M, Mandelkow E (2012). Biochemistry and cell biology of tau protein in neurofibrillary degeneration. Cold Spring Harbor Perspectives in Medicine.

[CR30] Mapstone M, Cheema AK, Fiandaca MS (2014). Plasma phospholipids identify antecedent memory impairment in older adults. Nature Medicine.

[CR31] Motsinger-Reif A, Zhu H, Kling MA (2013). Comparing metabolomic and pathologic biomarkers alone and in combination for discriminating Alzheimer’s disease from normal cognitive aging. Acta Neuropathologica Communications.

[CR32] Orešič M, Hyötyläinen T, Herukka S-K (2011). Metabolome in progression to Alzheimer’s disease. Translational Psychiatry.

[CR33] Perluigi M, Butterfield DA (2012). Oxidative Stress and Down Syndrome: A route toward Alzheimer-like dementia. Current Gerontology and Geriatrics Research.

[CR34] Perry G, Cash AD, Smith MA (2002). Alzheimer disease and oxidative stress. Journal of Biomedicine & Biotechnology.

[CR35] Pluskal T, Castillo S, Villar-Briones A (2010). MZmine 2: Modular framework for processing, visualizing, and analyzing mass spectrometry-based molecular profile data. BMC Bioinformatics.

[CR36] Rhein V, Song X, Wiesner A (2009). Amyloid-beta and tau synergistically impair the oxidative phosphorylation system in triple transgenic Alzheimer’s disease mice. PNAS.

[CR37] Roessner U, Bowne J (2009). What is metabolomics all about?. BioTechniques.

[CR38] Salek RM, Xia J, Innes A (2010). A metabolomic study of the CRND8 transgenic mouse model of Alzheimer’s disease. Neurochemistry International.

[CR39] Sato Y, Suzuki I, Nakamura T (2012). Identification of a new plasma biomarker of Alzheimer’s disease using metabolomics technology. Journal of Lipid Research.

[CR40] Tarawneh R, Holtzman DM (2012). The clinical problem of symptomatic Alzheimer’s disease and mild cognitive impairment. Cold Spring Harbor Perspectives in Medicine.

[CR41] Temmerman L, De Livera AM, Bowne JB (2013). Cross-platform urine metabolomics of experimental hyperglycemia in Type 2 diabetes. Journal of Diabetes & Metabolism.

[CR42] Trillo L, Das D, Hsieh W (2013). Ascending monoaminergic systems alterations in Alzheimer’s disease. Translating basic science into clinical care. Neuroscience and Biobehavioral Reviews.

[CR43] Trushina E, Dutta T, Persson X (2013). Identification of altered metabolic pathways in plasma and CSF in mild cognitive impairment and Alzheimer’s disease using metabolomics. PLoS One.

[CR44] Trushina E, Mielke MM (2013). Recent advances in the application of metabolomics to Alzheimer’s disease. Biochimica et Biophysica Acta.

[CR45] Wiltfang J, Esselmann H, Bibl M (2007). Amyloid beta peptide ratio 42/40 but not Abeta 42 correlates with phospho-Tau in patients with low- and high-CSF A beta 40 load. Journal of Neurochemistry.

[CR46] Xia J, Mandal R, Sinelnikov IV (2012). MetaboAnalyst 2.0: A comprehensive server for metabolomic data analysis. Nucleic acids Research.

[CR47] Yardim-Akaydin S, Sepici A, Ozkan Y (2006). Evaluation of allantoin levels as a new marker of oxidative stress in Behçet’s disease. Scandinavian Journal of Rheumatology.

[CR48] Zhang J-H (1999). A simple statistical parameter for use in evaluation and validation of high throughput screening assays. Journal of Biomolecular Screening.

[CR49] Zhao Y, Zhao B (2013). Oxidative stress and the pathogenesis of Alzheimer’s disease. Oxidative Medicine and Cellular Longevity.

[CR50] Zitnanová I, Korytár P, Aruoma OI (2004). Uric acid and allantoin levels in Down syndrome: Antioxidant and oxidative stress mechanisms?. Clinica Chimica Acta; International Journal of Clinical Chemistry.

