# Supplementary material for: Metabolic profiling of a transgenic Caenorhabditis elegans Alzheimer model
Source: Metabolomics. 2014 Jul 30;11(2):477–86. doi: 10.1007/s11306-014-0711-5 (PMC4342517; doi:10.1007/s11306-014-0711-5)
Supplement: Supplementary file 1 — Supplementary material 1 (DOCX 16892 kb) [file 11306_2014_711_MOESM1_ESM.docx]

**Supplemental information**

**Title**

Metabolic profiling of a transgenic *Caenorhabditis elegans* Alzheimer model


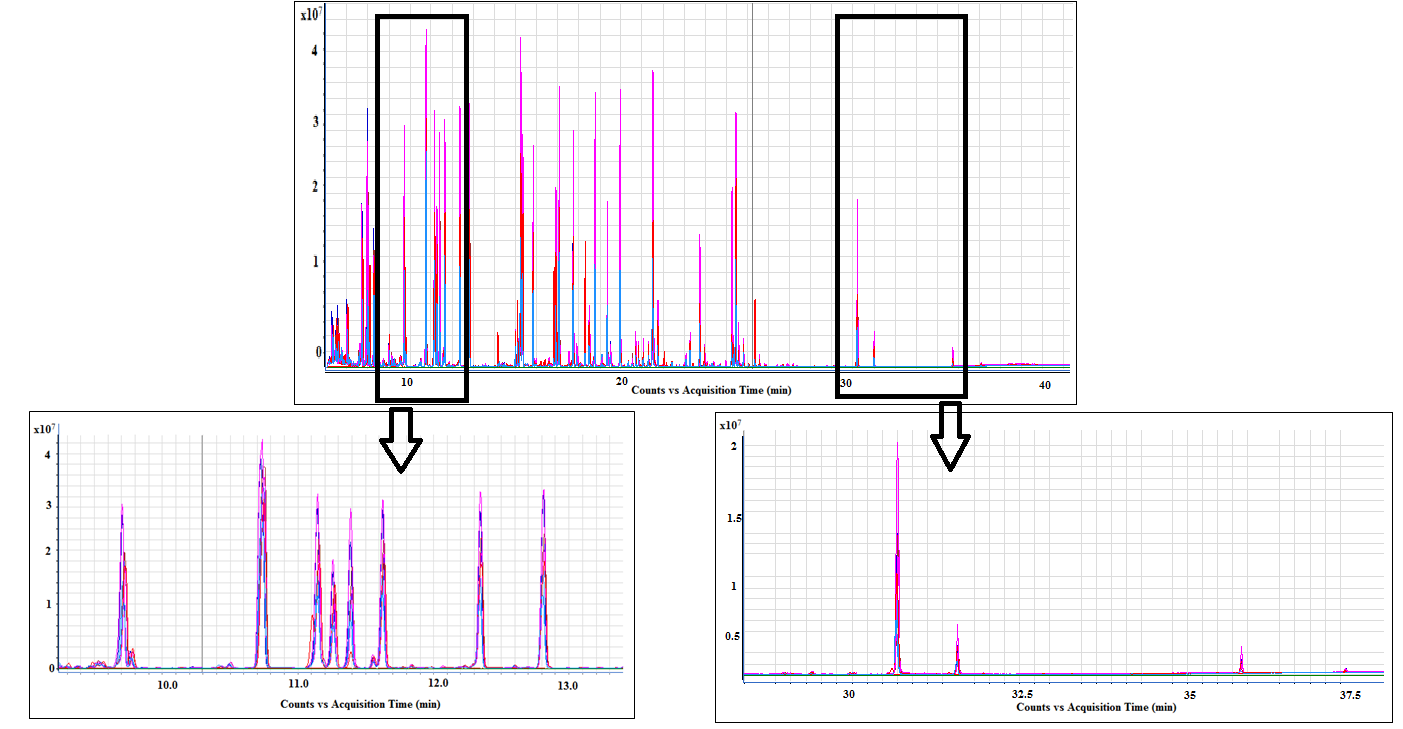


*Supplemental Fig. 1: Quality control process GC-MS. Every six samples, a pooled instrument control sample consisting of 54 standard compounds was run to evaluate potential retention time shifts and loss in sensitivity. All quality control runs overlapped clearly and no significant retetion time shift was present. A disturbing loss in sensitivity was not observed. It can therefore be taken into account by normalization strategies. X-axis: retention time, Y-axis: peak intensity.*


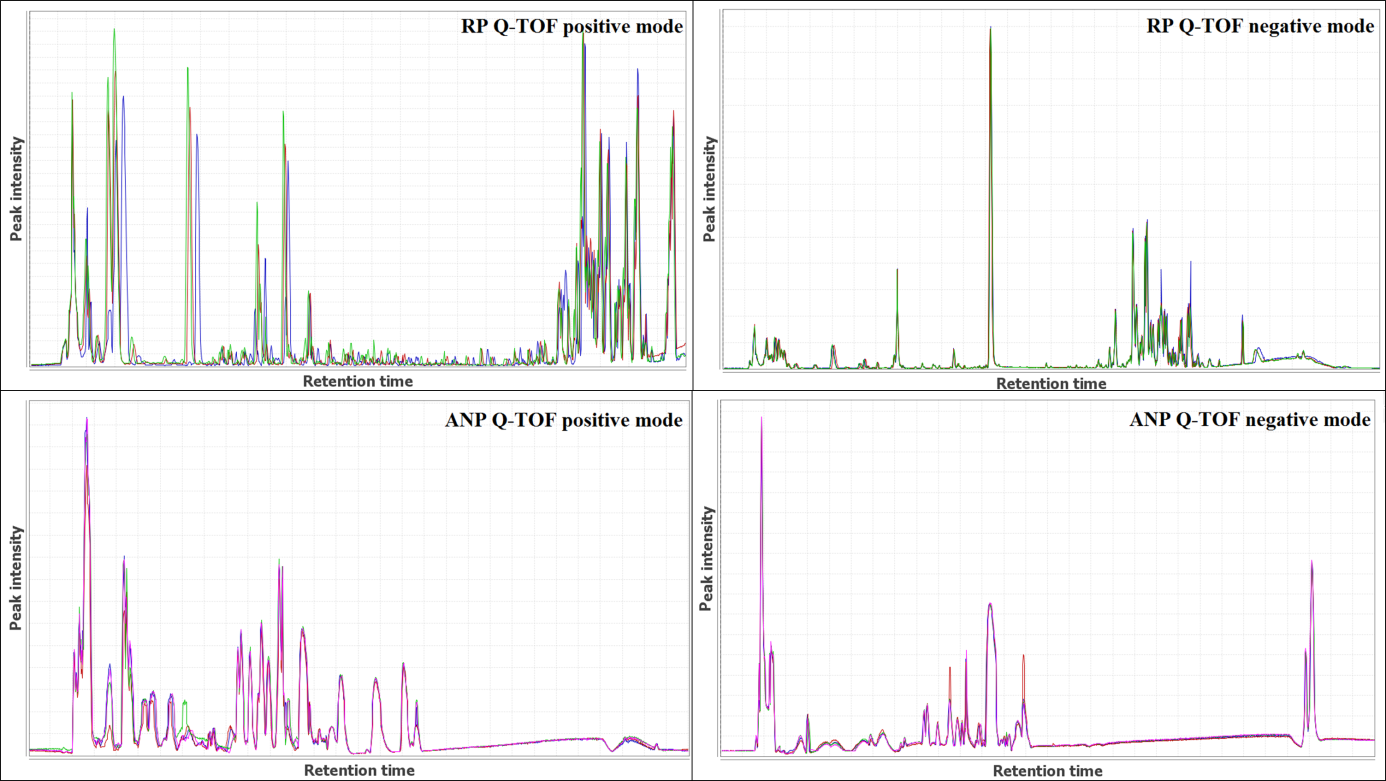


*Supplemental Fig. 2: Quality control runs for different LC-Q-TOF modes. Every 6 samples a pooled biological control sample was run to verify technical consistency. Retention time shifts were never bigger than 0.1 min. Mass accuracy was verified over time (always lower than 1.78 ppm), sensivity never dropped more than 15% and was uniform over runs, which is accounted for by normalization. X-axis: retention time (from 0-14 minutes), Y-axis: peak intensity (total ion count from 0 to 8.0E6).*


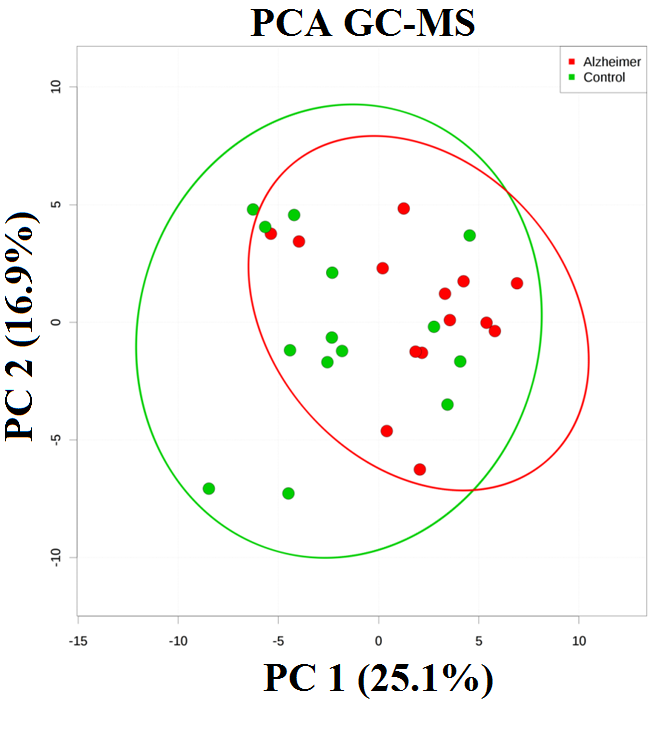


*Supplemental Fig. 3: Principal components analysis (PCA) plot of GC-MS data shows no clear separation of the control and Alzheimer groups. PCA plots were performed to evaluate the overall variance in the obtained datasets.*

*
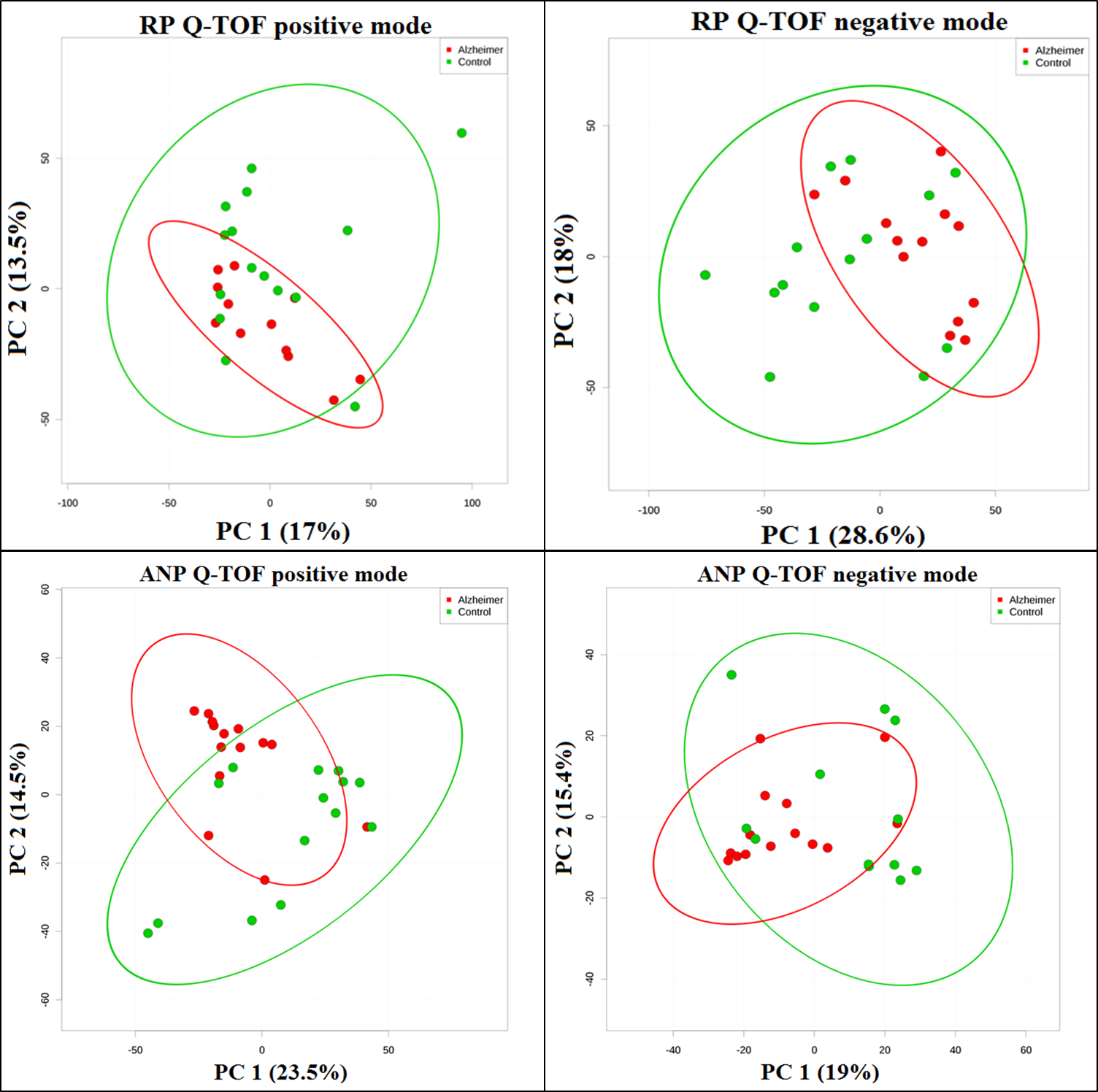
*

*Supplemental Fig. 4: PCA plots of LC-MS data show no clear separation of the control and Alzheimer groups. Red circles represent AD samples, green circles represent control samples. PCA plots were performed to evaluate the overall variance in the obtained datasets.*

*
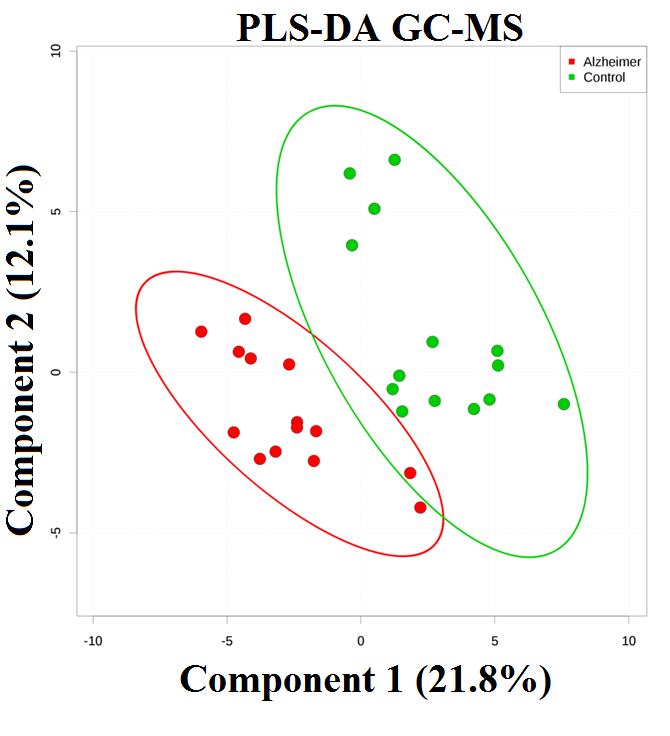
*

*Supplemental Fig. 5: The supervised partial-least squares discriminant analysis (PLS-DA) of GC-MS mode was conducted to better explore the variance differentiating the two experimental conditions (AD vs. control). In line with expectations, a better separation of the experimental groups was achieved.*

*
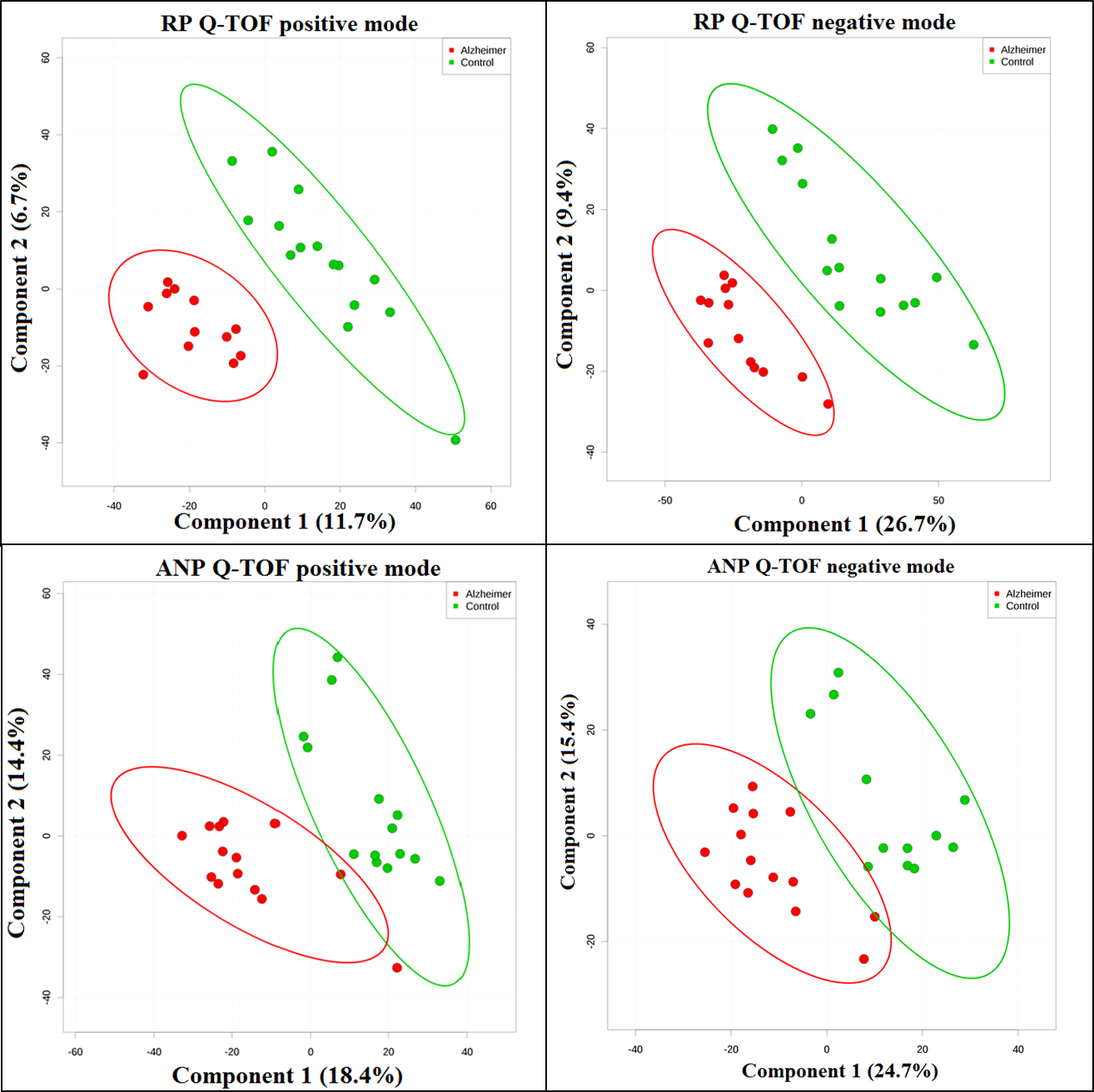
*

*Supplemental Fig. 6: The supervised partial-least squares discriminant analysis (PLS-DA) of LC-MS data was conducted to better explore the variance differentiating the two experimental conditions (AD vs. control). In line with expectations, a better separation of the experimental groups was achieved. Red circles represent AD samples, green circles represent control samples.*

*
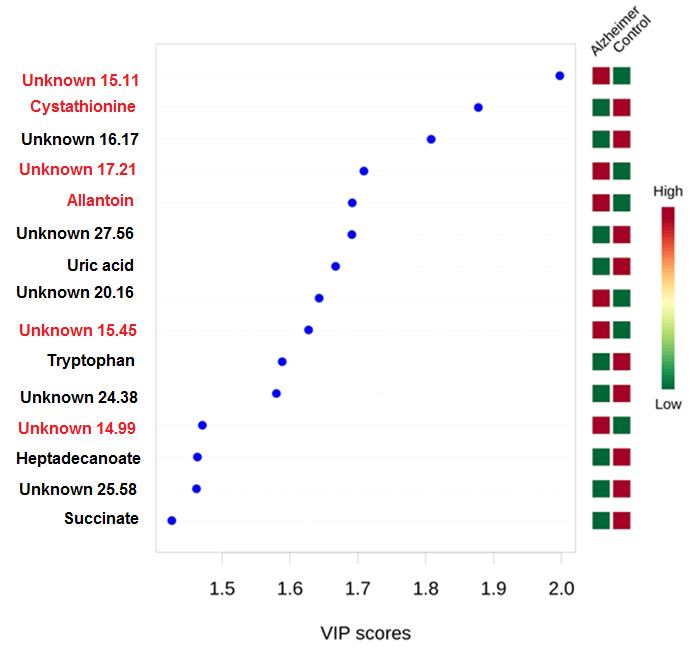
*

*Supplemental Fig. 7: Variable Importance in Projection (VIP) of GC-MS mode. VIP scores indicate the importance of a variable in projection using a PLS-DA model. VIP-scores > 1 can be considered as good variables in a given model. Differential features are highlighted in red. Retention time is given for the unidentified features. Red squares indicate upregulation of this feature in a condition, green squares indicate downregulation.*

*
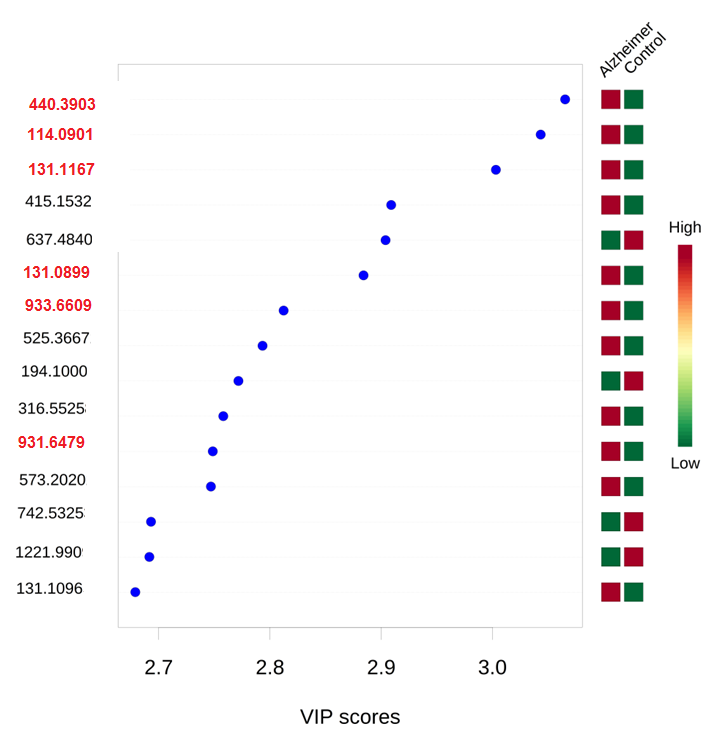
*

*Supplemental Fig. 8: Variable Importance in Projection (VIP) of RP-MS positive mode. VIP scores indicate the importance of a variable in projection using a PLS DA model. VIP-scores > 1 can be considered as good variables in a given model. Differential features are highlighted in red. Red squares indicate upregulation of this feature in a condition, green squares indicate downregulation.*

*
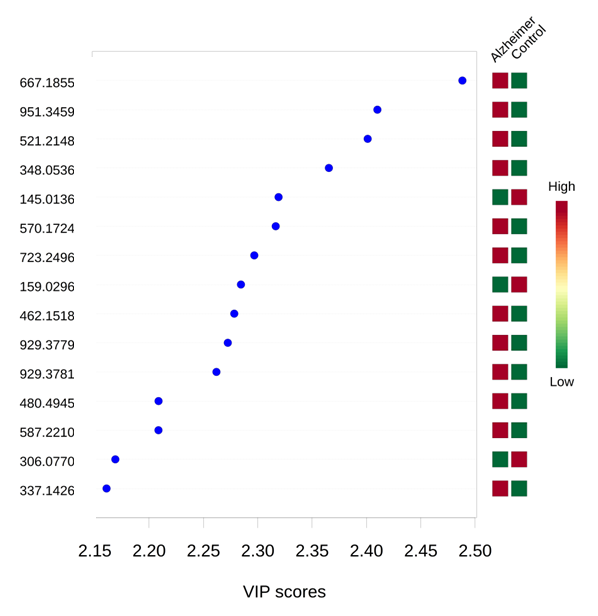
*

*Supplemental Fig. 9: Variable Importance in Projection (VIP) of RP-MS negative mode. VIP scores indicate the importance of a variable in projection using a PLS DA model. VIP-scores > 1 can be considered as good variables in a given model. All features shown are also differential. Red squares indicate upregulation of this feature in a condition, green squares indicate downregulation.
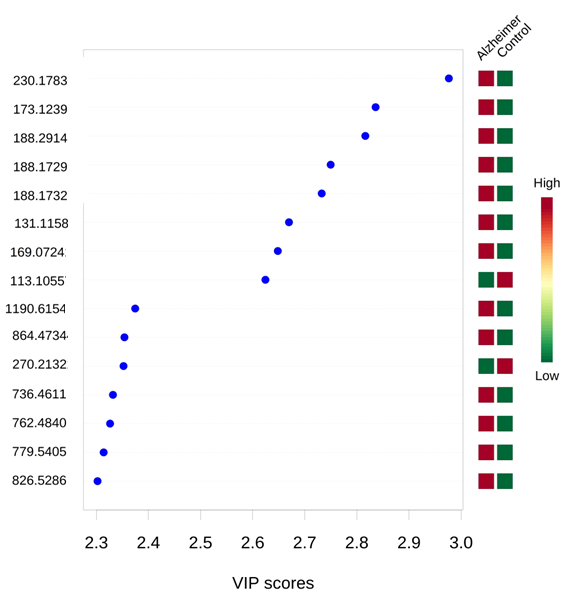
 Supplemental Fig. 10: Variable Importance in Projection (VIP) of ANP-MS positive mode. VIP scores indicate the importance of a variable in projection using a PLS DA model. VIP-scores > 1 can be considered as good variables in a given model. All features shown are also differential. Red squares indicate upregulation of this feature in a condition, green squares indicate downregulation.*

*
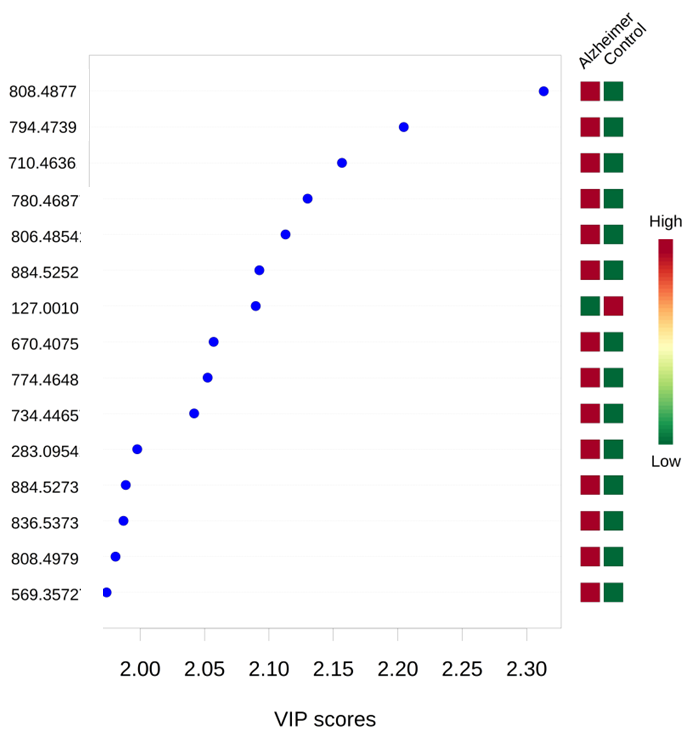
*

*Supplemental Fig. 11: Variable Importance in Projection (VIP) of ANP-MS negative mode. VIP scores indicate the importance of a variable in projection using a PLS DA model. VIP-scores > 1 can be considered as good variables in a given model. None of the features shown are differential. Red squares indicate upregulation of this feature in a condition, green squares indicate downregulation.*


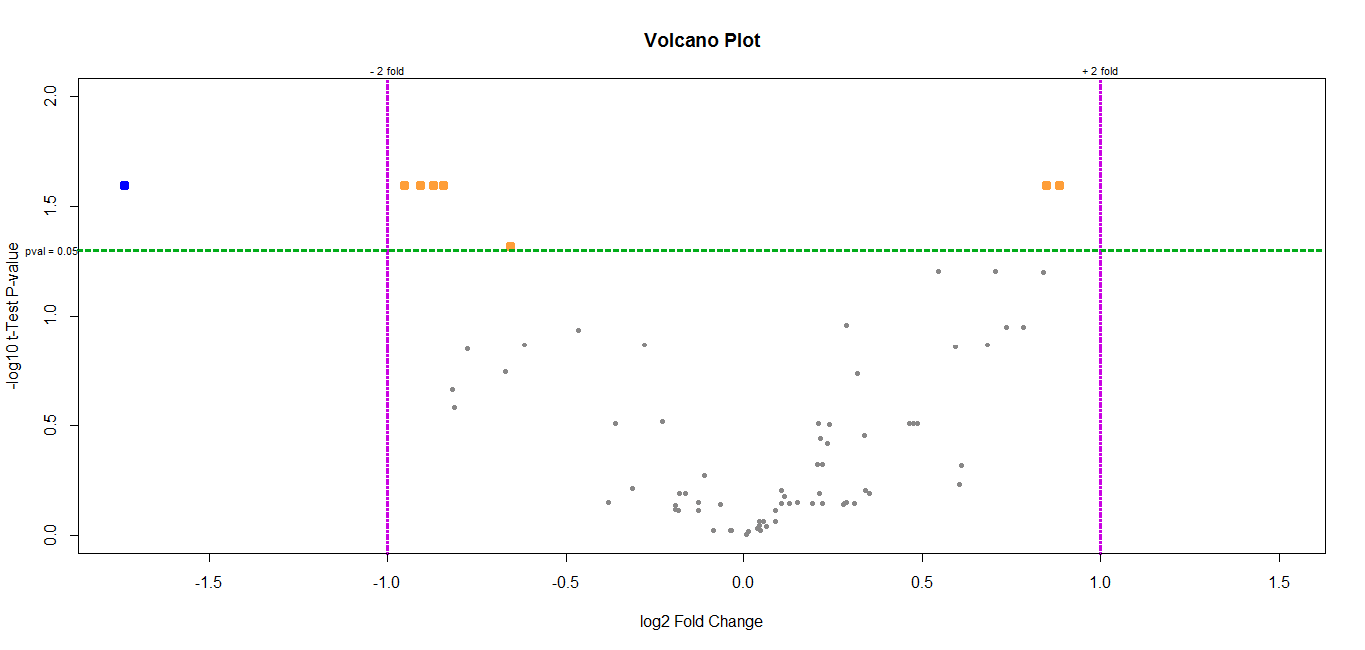


*Supplemental Fig. 12: Volcano plot from GC-MS mode. X-axis represents the fold change, ions with a fold change >±2 are selected. Y-axis represents the p value (adjusted according to the Benjamini & Hochberg principle), ions with p value<0.05 are selected. Red and blue dots represent ions which are respectively significantly upregulated and downregulated.*


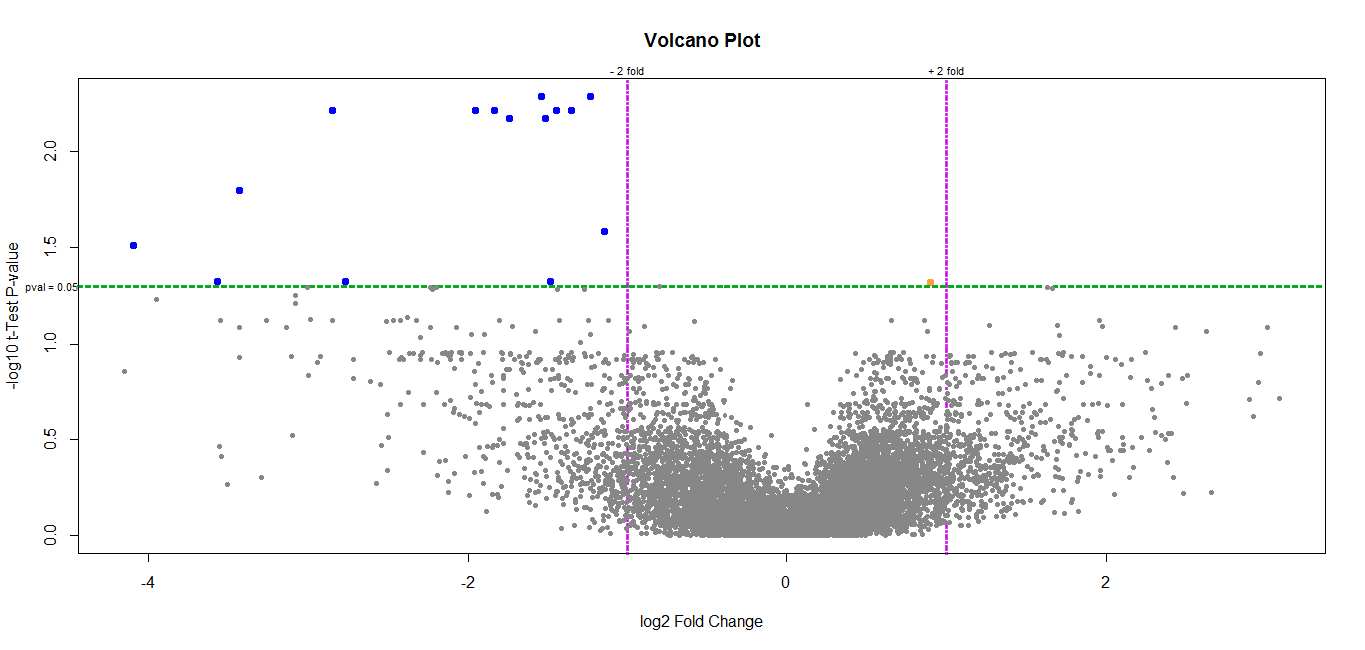


*Supplemental Fig. 13: Volcano plot from RP-MS positive mode. X-axis represents the fold change, ions with a fold change >±2 are selected. Y-axis represents the p value (adjusted according to the Benjamini & Hochberg principle), ions with p value<0.05 are selected. Red and blue dots represent ions which are respectively significantly upregulated and downregulated.*


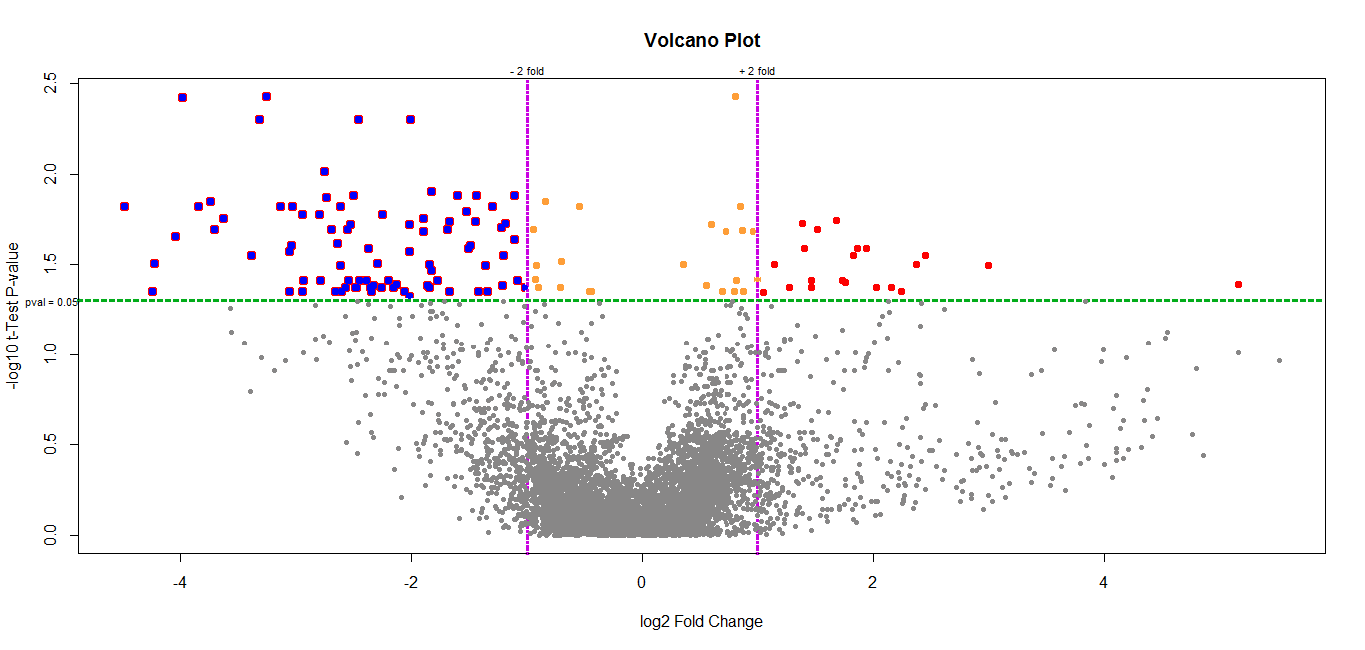


*Supplemental Fig. 14: Volcano plot from RP-MS negative mode. X-axis represents the fold change, ions with a fold change >±2 are selected. Y-axis represents the p value (adjusted according to the Benjamini & Hochberg principle), ions with p value<0.05 are selected. Red and blue dots represent ions which are respectively significantly upregulated and downregulated.*
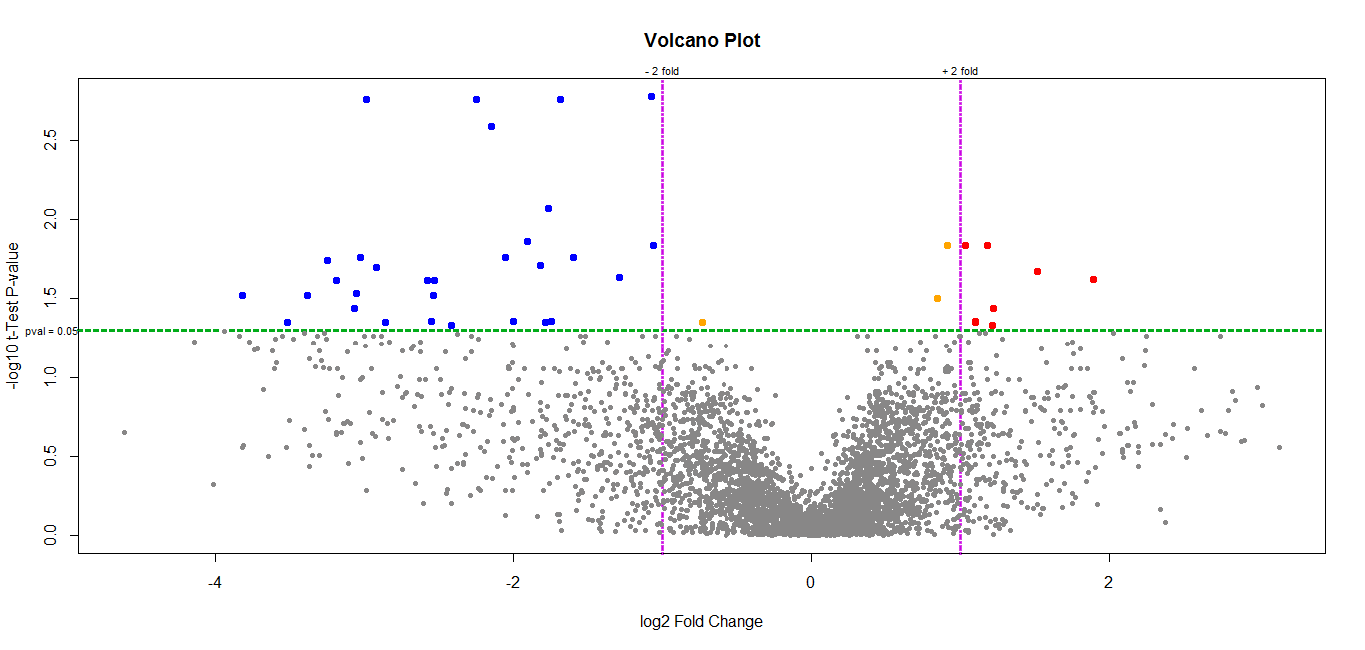


*Supplemental Fig. 15: Volcano plot from ANP-MS positive mode. X-axis represents the fold change, ions with a fold change >±2 are selected. Y-axis represents the p value, ions with p value<0.05 are selected. Red and blue dots represent ions which are respectively significantly upregulated and downregulated.*


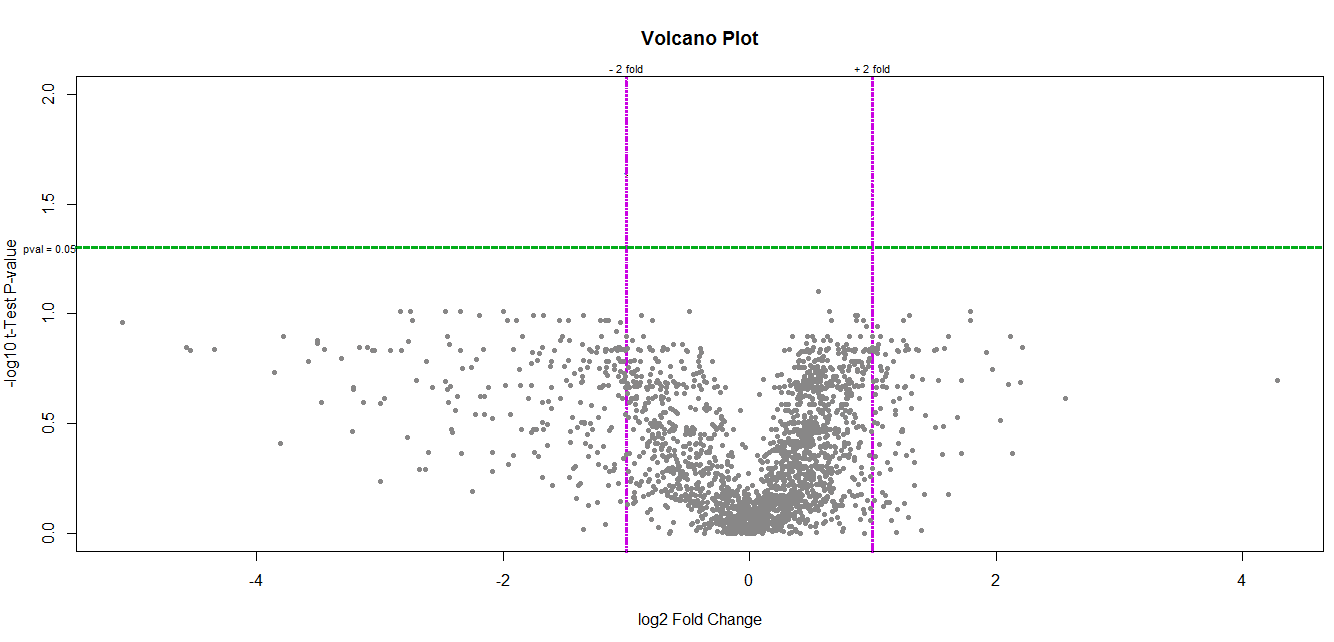


*Supplemental Fig. 16: Volcano plot from ANP-MS negative mode. X-axis represents the fold change, ions with a fold change >±2 are selected. Y-axis represents the p value (adjusted according to the Benjamini & Hochberg principle), ions with p value<0.05 are selected. Red and blue dots represent ions which are respectively significantly upregulated and downregulated.*

*
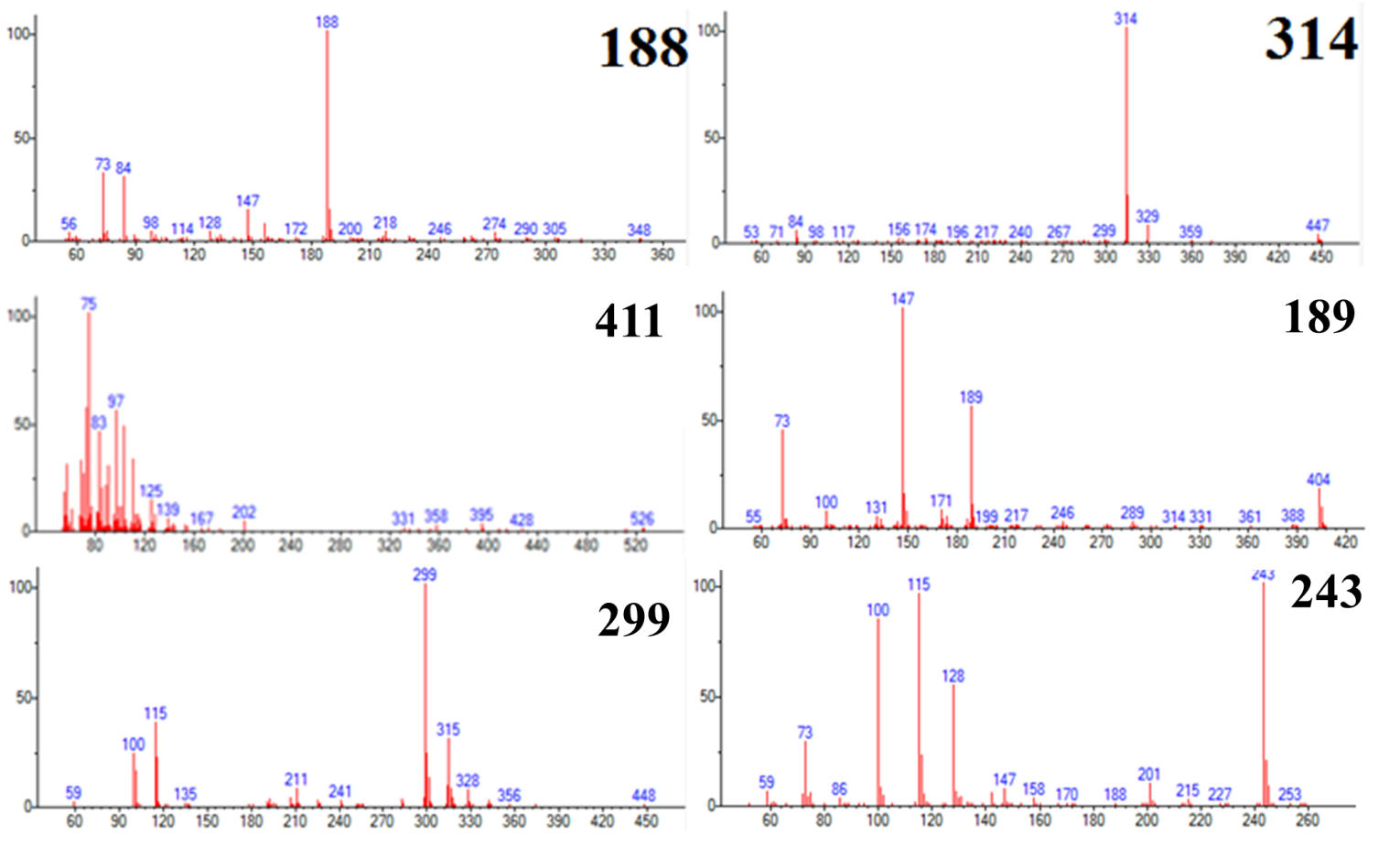
*

*Supplemental Fig. 17: Mass spectra of unknown differential GC-MS features. The number in the upper right corner corresponds to the mass identifiers.*


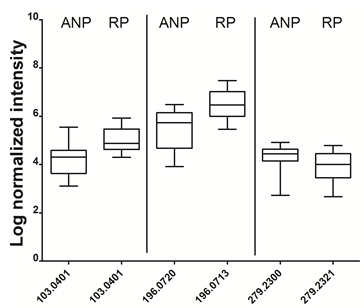


*Supplemental Fig. 18: Comparison of common features in ANP and RP modes in negative ion mode*

*Supplemental Table 1: Average mass accuracy deviation from internal standards in different LC-MS modes. This average deviation ensures the reproducibility over longer periods of running samples.*

| Average mass accuracy deviation | | |
| --- | --- | --- |
| Method | ion mode | Δppm |
| LC-MS:RP | + | 1.49 |
| LC-MS:RP | - | 1.25 |
| LC-MS:ANP | + | 1.78 |
| LC-MS:ANP | - | 0.32 |

*Supplemental Table 2: Mean and standard deviation of all samples in all modes.*

|  | **Method** | | | | | | | | | |
| --- | --- | --- | --- | --- | --- | --- | --- | --- | --- | --- |
| Sample | GC-MS | | ANP Q-TOF negative | | ANP Q-TOF positive | | RP Q-TOF negative | | RP Q-TOF positive | |
|  | Mean | Standard Deviation | Mean | Standard Deviation | Mean | Standard Deviation | Mean | Standard Deviation | Mean | Standard Deviation |
| Alzheimer 1 | 0.289218 | 2.193592 | -0.05821 | 2.434255 | 0.078918 | 2.387719 | 0.188092 | 2.313494 | -0.1192 | 2.589902 |
| Alzheimer 2 | 0.066625 | 2.431685 | 0.045026 | 2.338939 | 0.107688 | 2.422912 | 0.248602 | 2.248038 | -0.12889 | 2.525678 |
| Alzheimer 3 | 0.027113 | 2.150899 | -0.06346 | 2.573925 | 0.071822 | 2.468753 | 0.176934 | 2.45337 | -0.11701 | 2.487699 |
| Alzheimer 4 | 0.038008 | 2.073683 | 0.10108 | 2.440149 | 0.037176 | 2.525653 | 0.219542 | 2.39715 | -0.18061 | 2.587542 |
| Alzheimer 5 | -0.24049 | 2.233567 | 0.093079 | 2.452153 | -0.05332 | 2.761946 | 0.171522 | 2.449395 | -0.17355 | 2.541348 |
| Alzheimer 6 | 0.140631 | 2.127284 | 0.095642 | 2.514082 | 0.016102 | 2.686503 | 0.185053 | 2.483518 | -0.15573 | 2.614462 |
| Alzheimer 7 | -0.0091 | 2.15554 | -0.19676 | 2.749264 | -0.09665 | 2.70039 | 0.193727 | 2.367049 | -0.14612 | 2.527806 |
| Alzheimer 8 | -0.09595 | 1.886018 | -0.05752 | 2.536706 | -0.01936 | 2.597872 | 0.190346 | 2.431482 | -0.1707 | 2.596611 |
| Alzheimer 9 | -0.09273 | 2.244538 | -0.00544 | 2.521075 | 0.013408 | 2.57334 | x | x | -0.16053 | 2.528764 |
| Alzheimer 10 | 0.149282 | 2.422314 | -0.04272 | 2.756837 | -0.05756 | 2.740538 | 0.155196 | 2.426959 | -0.14313 | 2.578261 |
| Alzheimer 11 | 0.114088 | 2.17637 | -0.09886 | 2.639712 | 0.011105 | 2.612182 | 0.212485 | 2.408747 | -0.14173 | 2.599665 |
| Alzheimer 12 | -0.01567 | 2.288362 | -0.05453 | 2.51674 | 0.042291 | 2.514518 | 0.165994 | 2.42117 | -0.13226 | 2.534345 |
| Alzheimer 13 | 0.197501 | 2.148396 | -0.17502 | 2.846281 | -0.10136 | 2.928599 | 0.138847 | 2.543522 | x | x |
| Alzheimer 14 | 0.268837 | 2.233179 | -0.19133 | 2.965841 | -0.12945 | 2.856533 | 0.169206 | 2.576116 | -0.09104 | 2.556176 |
| Control 1 | 0.118745 | 2.085829 | 0.022472 | 2.924574 | -0.08687 | 2.781249 | 0.31142 | 3.008081 | -0.00045 | 2.66725 |
| Control 2 | 0.099936 | 2.203378 | -0.03341 | 2.724515 | -0.03485 | 2.749882 | 0.276245 | 2.602968 | -0.07955 | 2.555801 |
| Control 3 | 0.489007 | 2.065365 | -0.08008 | 2.70035 | 0.012973 | 2.55323 | 0.263156 | 2.509007 | -0.12766 | 2.609946 |
| Control 4 | 0.464009 | 2.299375 | -0.06268 | 2.749366 | -0.03541 | 2.759343 | 0.220962 | 2.461584 | -0.1352 | 2.604168 |
| Control 5 | -0.02621 | 2.383478 | 0.061814 | 2.66521 | -0.09455 | 2.843686 | 0.226446 | 2.607527 | -0.11119 | 2.610994 |
| Control 6 | 0.108725 | 2.107454 | 0.058593 | 2.648245 | -0.01464 | 2.77811 | 0.201352 | 2.435736 | -0.14075 | 2.536046 |
| Control 7 | -0.02777 | 2.475253 | 0.043967 | 2.911033 | 0.021338 | 2.778744 | 0.232835 | 2.833303 | 0.028768 | 2.645141 |
| Control 8 | 0.213967 | 2.150101 | 0.106957 | 2.696244 | 0.04575 | 2.578497 | 0.227922 | 2.722791 | 0.058015 | 2.643619 |
| Control 9 | -0.22721 | 2.354703 | -0.13964 | 3.160766 | -0.24699 | 3.116861 | 0.130245 | 2.549842 | -0.13243 | 2.555988 |
| Control 10 | -0.40613 | 2.839733 | x | x | -0.27489 | 3.170641 | 0.079321 | 2.4582 | -0.17093 | 2.52641 |
| Control 11 | 0.196368 | 2.330416 | x | x | 0.028957 | 2.624169 | 0.269518 | 2.668004 | 0.183354 | 2.752695 |
| Control 12 | 0.048334 | 2.098936 | 0.011495 | 2.813628 | -0.01154 | 2.688402 | 0.191693 | 2.665898 | -0.08764 | 2.574055 |
| Control 13 | -0.0174 | 2.315875 | -0.26465 | 2.939879 | -0.15786 | 2.834979 | 0.169733 | 2.529776 | -0.09384 | 2.56488 |
| Control 14 | 0.152169 | 2.369799 | -0.20765 | 3.044097 | -0.25134 | 3.04504 | 0.154241 | 2.64557 | -0.04589 | 2.546521 |

*Supplemental Table 3: Significant differential features combined from different modes. Mass, retention time, p value (*adjusted according to the Benjamini & Hochberg principle), fold change, ion mode and putative confirmation of those metabolites in literature are shown. Adducts indicated with same superscript number.*

| **LC-MS differential features** | | | | | |
| --- | --- | --- | --- | --- | --- |
| Mass (m/z) | RT | *p* value* | Fold change | Mode | Putative confirmation literature |
| 173.1108 | 3.1 | *0.0017* | 2.11 | ANP+ |  |
| 131.1159 | 7.0 | *0.0017* | 4.76 | ANP+ |  |
| 169.0724 | 7.0 | *0.0017* | 3.21 | ANP+ |  |
| 230.1783 | 8.1 | *0.0017* | 7.92 | ANP+ |  |
| 131.1159 | 7.1 | *0.0026* | 4.42 | ANP+ |  |
| 384.1027 | 1.4 | *0.0037* | 0.57 | RP- |  |
| 509.3433 | 12.4 | *0.0037* | 9.54 | RP- | Lin et al. 2013 |
| 929.6274^1^ | 11.6 | *0.0038* | 15.77 | RP- |  |
| 366.1274 | 12.7 | *0.0050* | 9.96 | RP- |  |
| 929.8786 | 3.8 | *0.0050* | 4.03 | RP- |  |
| 464.5713^1^ | 11.6 | *0.0050* | 5.48 | RP- |  |
| 114.0901 | 0.8 | *0.0052* | 2.91 | RP+ |  |
| 448.2284 | 9.7 | *0.0052* | 2.35 | RP+ |  |
| 131.1167 | 0.8 | *0.0061* | 3.56 | RP+ |  |
| 362.2059 | 8.8 | *0.0061* | 2.56 | RP+ |  |
| 131.2164 | 0.8 | *0.0061* | 2.72 | RP+ |  |
| 485.1334 | 4.4 | *0.0061* | 7.21 | RP+ |  |
| 516.0773 | 3.7 | *0.0061* | 3.87 | RP+ |  |
| 131.0899 | 0.8 | *0.0068* | 2.85 | RP+ |  |
| 397.2089 | 8.5 | *0.0068* | 3.35 | RP+ |  |
| 173.1239 | 3.0 | *0.0084* | 3.39 | ANP+ |  |
| 449.1221 | 8.0 | *0.0096* | 6.72 | RP- |  |
| 929.3781 | 3.9 | *0.0125* | 3.54 | RP- |  |
| 219.0865 | 2.3 | *0.0131* | 5.67 | RP- |  |
| 386.1452 | 4.7 | *0.0131* | 2.16 | RP- | Trushina et al. 2013 |
| 721.1036 | 3.5 | *0.0131* | 3.03 | RP- |  |
| 559.1440 | 7.6 | *0.0131* | 2.71 | RP- |  |
| 482.3108 | 11.2 | *0.0134* | 6.65 | RP- |  |
| 188.2915 | 11.3 | *0.0138* | 3.75 | ANP+ |  |
| 466.3274^1^ | 11.6 | *0.0141* | 13.30 | RP- |  |
| 337.1427 | 11.2 | *0.0141* | 1.79 | RP- |  |
| 505.1993 | 5.8 | *0.0145* | 0.53 | ANP+ |  |
| 409.0493 | 6.4 | *0.0145* | 0.49 | ANP+ |  |
| 549.1047 | 6.4 | *0.0145* | 0.44 | ANP+ |  |
| 120.0793 | 2.8 | *0.0145* | 2.09 | ANP+ |  |
| 961.6186^2^ | 11.3 | *0.0150* | 22.27 | RP- |  |
| 914.5292 | 10.0 | *0.0150* | 6.12 | RP- |  |
| 787.2068 | 5.3 | *0.0150* | 2.46 | RP- |  |
| 449.1218 | 7.6 | *0.0150* | 8.17 | RP- |  |
| 608.1493 | 8.9 | *0.0150* | 0.55 | RP- |  |
| 582.3737^3^ | 11.8 | *0.0150* | 8.75 | RP- |  |
| 480.4946^2^ | 11.3 | *0.0150* | 14.31 | RP- |  |
| 347.1713 | 11.2 | *0.0151* | 1.46 | RP- | Lin et al. 2013 |
| 650.2202 | 7.2 | *0.0160* | 10.80 | RP+ |  |
| 348.0537 | 1.0 | *0.0161* | 2.88 | RP- |  |
| 951.3460 | 6.3 | *0.0166* | 4.76 | RP- |  |
| 886.5142 | 12.7 | *0.0166* | 6.94 | RP- |  |
| 755.2796 | 6.1 | *0.0166* | 7.70 | RP- |  |
| 188.1732 | 11.6 | *0.0174* | 3.03 | ANP+ |  |
| 858.4997 | 5.4 | *0.0174* | 4.16 | ANP+ |  |
| 840.4818 | 7.4 | *0.0174* | 8.13 | ANP+ |  |
| 566.2537 | 11.6 | *0.0174* | 12.36 | RP- |  |
| 607.2028 | 7.2 | *0.0174* | 3.73 | RP- |  |
| 902.5655 | 2.7 | *0.0180* | 9.49 | ANP+ |  |
| 454.2554 | 8.6 | *0.0180* | 0.31 | RP- |  |
| 376.1198 | 4.5 | *0.0182* | 3.19 | RP- |  |
| 525.1274 | 4.6 | *0.0182* | 2.73 | RP- |  |
| 618.2788 | 10.0 | *0.0187* | 0.38 | RP- |  |
| 339.1420 | 11.2 | *0.0187* | 2.28 | RP- | Trushina et al. 2013 |
| 271.2258 | 12.0 | *0.0190* | 0.66 | RP- |  |
| 521.2149 | 6.3 | *0.0190* | 5.78 | RP- |  |
| 220.0761 | 11.5 | *0.0190* | 4.04 | RP- |  |
| 188.1730 | 11.0 | *0.0193* | 3.53 | ANP+ |  |
| 627.3304 | 9.8 | *0.0196* | 2.33 | RP- |  |
| 828.5257 | 6.0 | *0.0199* | 7.57 | ANP+ |  |
| 667.1847 | 3.9 | *0.0201* | 1.93 | RP- |  |
| 359.0209 | 3.4 | *0.0201* | 0.35 | RP- |  |
| 482.3122 | 11.3 | *0.0201* | 5.86 | RP- |  |
| 494.3246 | 11.7 | *0.0201* | 13.04 | RP- |  |
| 929.3780 | 3.8 | *0.0201* | 3.22 | RP- |  |
| 466.3256 | 11.8 | *0.0201* | 6.48 | RP- |  |
| 543.2429 | 10.1 | *0.0204* | 0.55 | RP- |  |
| 667.1856 | 3.5 | *0.0208* | 3.73 | RP- |  |
| 614.3279 | 9.4 | *0.0208* | 0.61 | RP- |  |
| 493.1857 | 9.1 | *0.0208* | 0.51 | RP- |  |
| 270.2132 | 5.8 | *0.0212* | 0.35 | ANP+ |  |
| 1056.3666 | 7.3 | *0.0219* | 16.43 | RP- |  |
| 350.0537 | 1.3 | *0.0228* | 2.16 | RP- |  |
| 856.4912 | 5.0 | *0.0232* | 2.45 | ANP+ |  |
| 113.1056 | 11.2 | *0.0238* | 0.27 | ANP+ |  |
| 702.3237 | 8.0 | *0.0241* | 6.23 | RP- |  |
| 671.3998 | 5.5 | *0.0241* | 2.29 | ANP- |  |
| 824.4982 | 7.7 | *0.0242* | 5.98 | ANP+ |  |
| 824.5092 | 7.5 | *0.0242* | 5.77 | ANP+ |  |
| 1012.5129 | 6.2 | *0.0242* | 9.13 | ANP+ |  |
| 723.2497 | 7.2 | *0.0246* | 2.80 | RP- |  |
| 494.3233^4^ | 11.5 | *0.0246* | 8.19 | RP- |  |
| 659.1859 | 4.1 | *0.0257* | 2.85 | RP- |  |
| 559.0438^5^ | 0.7 | *0.0258* | 0.38 | RP- |  |
| 464.3112 | 11.7 | *0.0258* | 5.17 | RP- |  |
| 159.0297 | 1.2 | *0.0258* | 0.26 | RP- |  |
| 199.0365 | 0.9 | *0.0258* | 0.27 | RP- |  |
| 342.1580 | 4.5 | *0.0259* | 2.22 | RP+ |  |
| 480.3387^3^ | 11.8 | *0.0266* | 8.33 | RP- |  |
| 626.3630 | 11.3 | *0.0266* | 4.05 | RP- |  |
| 306.0758 | 0.8 | *0.0280* | 0.18 | RP- |  |
| 101.0243 | 1.0 | *0.0280* | 0.28 | RP- |  |
| 438.4403 | 10.0 | *0.0280* | 10.43 | RP- |  |
| 490.3248 | 11.1 | *0.0280* | 2.30 | RP- |  |
| 840.4828 | 7.2 | *0.0293* | 8.30 | ANP+ |  |
| 849.5176 | 6.2 | *0.0299* | 10.43 | ANP+ |  |
| 436.2475 | 6.5 | *0.0299* | 5.81 | ANP+ |  |
| 1190.6154 | 6.1 | *0.0299* | 14.09 | ANP+ |  |
| 328.0459 | 1.6 | *0.0303* | 1.63 | RP- |  |
| 933.6609 | 11.6 | *0.0307* | 17.07 | RP+ |  |
| 931.6426 | 11.6 | *0.0311* | 18.71 | RP- |  |
| 478.3177 | 11.3 | *0.0311* | 4.91 | RP- |  |
| 298.1658 | 6.4 | *0.0314* | 0.55 | ANP+ |  |
| 461.1953 | 9.3 | *0.0314* | 0.78 | RP- |  |
| 548.2950 | 11.3 | *0.0314* | 3.60 | RP- |  |
| 658.2943 | 9.4 | *0.0314* | 0.45 | RP- |  |
| 487.1361 | 0.8 | *0.0314* | 0.19 | RP- |  |
| 597.0578 | 4.6 | *0.0317* | 1.89 | RP- |  |
| 780.3302 | 9.1 | *0.0317* | 0.13 | RP- |  |
| 462.1519 | 5.7 | *0.0317* | 6.11 | RP- |  |
| 572.3035 | 9.3 | *0.0317* | 2.56 | RP- |  |
| 496.3387^4^ | 11.5 | *0.0342* | 3.54 | RP- |  |
| 660.1303 | 6.4 | *0.0364* | 0.43 | ANP+ |  |
| 848.5050 | 6.0 | *0.0364* | 8.37 | ANP+ |  |
| 626.3276 | 10.0 | *0.0379* | 1.90 | RP- |  |
| 874.3086 | 8.2 | *0.0379* | 0.50 | RP- |  |
| 199.0354 | 0.9 | *0.0385* | 0.30 | RP- |  |
| 580.2341^6^ | 11.2 | *0.0385* | 4.58 | RP- |  |
| 541.1447 | 0.7 | *0.0385* | 0.36 | RP- |  |
| 360.2733 | 10.8 | *0.0385* | 0.57 | RP- |  |
| 987.6298 | 10.7 | *0.0385* | 5.82 | RP- |  |
| 640.2898^7^ | 11.4 | *0.0385* | 3.42 | RP- | Lin et al. 2013 |
| 514.0620 | 3.7 | *0.0385* | 6.89 | RP- |  |
| 554.3787 | 11.7 | *0.0385* | 7.65 | RP- |  |
| 478.3222 | 12.2 | *0.0385* | 5.45 | RP- |  |
| 570.3637^7^ | 11.4 | *0.0385* | 5.23 | RP- |  |
| 215.1592 | 0.7 | *0.0388* | 2.12 | RP- |  |
| 313.0481 | 1.0 | *0.0396* | 0.30 | RP- |  |
| 760.2077 | 5.3 | *0.0406* | 4.38 | RP- |  |
| 831.5874 | 11.8 | *0.0406* | 0.03 | RP- |  |
| 938.3802 | 3.2 | *0.0412* | 2.32 | RP- |  |
| 587.2211 | 4.6 | *0.0412* | 3.63 | RP- |  |
| 526.0336 | 3.9 | *0.0412* | 0.68 | RP- |  |
| 623.1998 | 6.2 | *0.0412* | 5.01 | RP- |  |
| 478.3202 | 12.0 | *0.0421* | 5.11 | RP- |  |
| 480.3091^2^ | 11.3 | *0.0421* | 5.94 | RP- |  |
| 596.1314 | 1.3 | *0.0421* | 2.02 | RP- |  |
| 180.2259 | 1.3 | *0.0421* | 1.64 | RP- |  |
| 481.3090^6^ | 11.2 | *0.0421* | 4.79 | RP- |  |
| 341.0660 | 4.4 | *0.0421* | 0.36 | RP- |  |
| 886.5147 | 12.6 | *0.0421* | 3.59 | RP- |  |
| 481.2605 | 11.3 | *0.0421* | 0.25 | RP- |  |
| 997.5264 | 10.0 | *0.0421* | 4.45 | RP- |  |
| 524.2846 | 11.6 | *0.0421* | 5.11 | RP- |  |
| 558.2669 | 8.3 | *0.0421* | 0.41 | RP- |  |
| 556.0887 | 4.6 | *0.0421* | 0.22 | RP- |  |
| 398.1538 | 11.2 | *0.0421* | 1.86 | RP- |  |
| 945.5858 | 10.8 | *0.0421* | 5.59 | RP- |  |
| 506.1160 | 5.6 | *0.0422* | 5.57 | RP- |  |
| 520.0780 | 6.4 | *0.0440* | 0.47 | ANP+ |  |
| 762.4840 | 4.5 | *0.0440* | 4.00 | ANP+ |  |
| 736.4611 | 4.5 | *0.0440* | 3.36 | ANP+ |  |
| 864.4734 | 6.1 | *0.0440* | 5.85 | ANP+ |  |
| 180.0657 | 1.3 | *0.0446* | 1.36 | RP- |  |
| 265.1475 | 11.1 | *0.0446* | 0.57 | RP- |  |
| 524.2976 | 13.9 | *0.0446* | 3.19 | RP- |  |
| 465.1534^8^ | 8.2 | *0.0446* | 0.62 | RP- |  |
| 354.0735 | 0.7 | *0.0446* | 1.38 | RP- |  |
| 492.3428 | 11.8 | *0.0446* | 6.32 | RP- |  |
| 650.2569 | 6.0 | *0.0446* | 6.07 | RP- |  |
| 166.0194^5^ | 0.7 | *0.0446* | 18.94 | RP- |  |
| 480.3403 | 12.2 | *0.0446* | 8.32 | RP- |  |
| 897.2883 | 7.5 | *0.0446* | 5.08 | RP- |  |
| 592.3576 | 11.0 | *0.0446* | 2.54 | RP- |  |
| 495.3247 | 11.7 | *0.0446* | 7.67 | RP- |  |
| 668.2849^7^ | 11.4 | *0.0446* | 4.18 | RP- |  |
| 405.2181 | 5.8 | *0.0446* | 2.68 | RP- | Lin et al. 2013 |
| 466.1571^8^ | 8.2 | *0.0446* | 0.54 | RP- |  |
| 653.1407 | 1.0 | *0.0446* | 0.21 | RP- |  |
| 162.8385 | 10.3 | *0.0447* | 0.48 | RP- |  |
| 438.0753 | 6.4 | *0.0447* | 0.47 | ANP+ |  |
| 931.5152 | 6.3 | *0.0447* | 11.43 | ANP+ |  |
| 798.4845 | 5.8 | *0.0447* | 3.44 | ANP+ |  |
| 975.5311 | 6.4 | *0.0447* | 7.25 | ANP+ |  |
| 551.3549 | 1.5 | *0.0447* | 1.66 | ANP+ |  |
| 244.1892 | 1.7 | *0.0467* | 0.43 | ANP+ |  |
| 850.5135 | 8.0 | *0.0467* | 5.34 | ANP+ |  |
| 632.1628 | 5.3 | *0.0473* | 6.80 | RP+ |  |
| 822.6104 | 11.4 | *0.0474* | 4.06 | RP- |  |
| 440.3903 | 10.7 | *0.0475* | 2.80 | RP+ |  |
| 931.6479 | 11.6 | *0.0475* | 11.86 | RP+ |  |
| 161.1369 | 0.3 | *0.0477* | 0.54 | RP+ |  |

*Supplemental Table 4: Accurate mass of tyrosine using Q Exactive Hybrid Quadrupole-Orbitrap mass spectrometer. Accurate mass (m/z value), p value (*adjusted according to the Benjamini & Hochberg principle), fold change, ion mode, Δppm and identification are shown.*

| **Accurate mass differential features LC-MS** | | | | | | |
| --- | --- | --- | --- | --- | --- | --- |
| *Mass (m/z)* | *Accurate mass* | *p* value* | Fold change | Mode | Δppm | Identification |
| 180.065 | 180.0665 | *0.0446* | 1.36 | RP- | 0 | Tyrosine |
